# Scaling Up a Diabetes Prevention Program in Geographically and Ethnoculturally Diverse Urban Regions of Canada: Protocol for a Hybrid Type 2 Implementation-Effectiveness Study

**DOI:** 10.2196/80276

**Published:** 2026-01-19

**Authors:** Katie Weatherson, Jessica E Bourne, Kaela Cranston, Kyra Braaten, Natalie Grieve, Joseph Kelly, Mary E Jung

**Affiliations:** 1 Department of Health and Exercise Sciences University of British Columbia Okanagan Kelowna, BC Canada

**Keywords:** diabetes prevention program, implementation science, implementation effectiveness study, sustainability

## Abstract

**Background:**

It is estimated that type 2 diabetes (T2D) impacts an estimated 5.3 million Canadians, despite the condition being largely preventable. Laboratory-based diabetes prevention programs (DPPs) have limited effectiveness when translated into community settings due to their low-quality delivery and inability to reach people in the community most in need. To date, no community-based DPPs have been implemented nationwide across Canada. Small Steps for Big Changes (SSBC) is a diet and physical activity counseling intervention that significantly reduces the risk of developing T2D and has been designed for feasible delivery by community-dwelling peers. To ensure maximum public health impact, SSBC must be optimally implemented, demonstrate effectiveness for diverse groups, and be sustainable over time.

**Objective:**

This project aims to adapt SSBC and evaluate the implementation, effectiveness, and sustainability of SSBC in diverse urban communities across Canada.

**Methods:**

A hybrid type 2 implementation-effectiveness study design using multiple and mixed methods will be used to evaluate the implementation and effectiveness of SSBC over 6 years in partnership with 11 regional Young Men’s Christian Associations across 8 provinces in Canada. Beginning in 2024, we will (1) adapt and implement SSBC in diverse urban cities across Canada; (2) examine the implementation (including implementation strategies), effectiveness, and cost-effectiveness of SSBC (2024-2028); and (3) determine the sustainability of SSBC at each delivery location (2028-2029). Data will be collected from SSBC clients, coaches, site leads, and senior leadership municipality partners. The project will be overseen by an advisory group and 3 committees focused on sex, gender, and inclusivity; program evaluation; and diabetes prevention engagement. This study has received ethical approval from the University of British Columbia Clinical Research Ethics Board (H23-01930).

**Results:**

Funding for this project began in October 2022, and Institutional Review Board approval was obtained in October 2023. Program implementation within each region is occurring in a phased approach, with partners beginning program delivery in one site (2024-2025) before expanding to any additional locations (2025-2027). Program enrollment occurs continuously during the implementation phase across all sites. From 2024-2025, a total of 13 delivery sites began program delivery, 722 participants have enrolled in the program, and 406 have begun the program (153/342, 44.7% non-Western or Eastern European; 80/345, 23.2% men or 82/347, 23.6% male). An additional 13 sites have confirmed they will launch the program in 2026.

**Conclusions:**

This study will demonstrate that SSBC can be scaled up nationwide to effectively and equitably reduce the Canadian population-level risk of T2D. This work will determine best practice implementation determinants, outcomes, and strategies critical for sustaining DPP implementation across Canada and beyond. Project findings will be shared with municipality partners and will be copresented with partners and SSBC clients to community organizations, local interested parties, and academics.

**Trial Registration:**

ClinicalTrials.gov NCT06440395; https://clinicaltrials.gov/study/NCT06440395

**International Registered Report Identifier (IRRID):**

DERR1-10.2196/80276

## Introduction

The incidence of type 2 diabetes (T2D) is continuing to rise worldwide [1,2] despite overwhelming evidence that this chronic disease is largely preventable [3-5]. As the fastest-growing chronic condition, T2D impacts an estimated 5.3 million Canadians [6], with estimated annual treatment costs of CAD $30 billion (a currency exchange rate of CAD $1=US $0.71 was applicable) [7]. An additional 6 million Canadians [6] live with prediabetes, a clinical condition recognized by the World Health Organization that precedes T2D. Further, 70% of individuals with prediabetes are estimated to progress to T2D in the next 10 years [8]. Rates of T2D are higher in areas with lower household income and education level, higher levels of food insecurity, unemployment, immigrant status, and among individuals of African, South Asian, and Indigenous origins [7-10]. Racial, sex, and gender-based health inequities in accessing care exacerbate these barriers [11-16]. Preventing the progression of prediabetes to T2D and addressing health inequities is a public health priority in Canada [17].

T2D prevention programs (diabetes prevention program [DPPs]) have been shown to reduce the progression of prediabetes to T2D in diverse populations and geographic regions of the world. Three landmark trials involving more than 4000 individuals with prediabetes demonstrated that increasing physical activity (PA) and inducing modest weight loss through dietary intervention can reduce the risk of developing T2D by between 50% to 60% [18-20]. Notably, diet and PA approaches were twice as efficacious as pharmaceutical interventions [18]. However, translating these highly efficacious DPPs into community settings for public health impact often results in reduced effectiveness [21,22]. This drop in efficacy is often attributed to inequitable access [23,24], poor fidelity [25-29], lack of cultural tailoring [11,30-34], and lack of sustainability plans [30,35-37].

To date, no community-based nationwide DPP has been implemented across Canada. To make a measurable impact on T2D prevention at a population level, potent evidence-based interventions must be implemented effectively at scale. Nationwide uptake of DPPs is hindered by the absence of evidence-informed implementation plans tailored to local contexts [38] and the examination of individual- and between-delivery site differences to understand the impact of context on implementation. Site contextual differences are common, as each community has unique challenges and opportunities that impact implementation, and remain an understudied paradox of implementation efforts [39]. Additionally, equity-based contextual factors, including access and cultural relevance for populations traditionally underserved and racialized against, are pivotal considerations for implementation scale-up [40,41]. It is imperative to disaggregate multisite data and understand how community-, organization-, provider-, and client-level factors contribute to implementation outcomes when organizations deliver the same program in different contexts [41,42]. Implementation studies with a focus on context, and in particular health equity, are needed to compare within and between-site implementation outcomes and determinants [41]. Understanding these contextual factors is also key for identifying methods (implementation strategies) required to enhance the implementation and scale-up of DPPs [43].

Further limiting DPPs from achieving population-level health impact is a failure to prioritize and support the sustainability of these evidence-based programs after they have been implemented. Sustainability, defined as the continued use of program components at sufficient intensity for the sustained achievement of desirable program goals and population outcomes [44], is now considered “one of the most significant translational research problems of our time” [45]. Sustaining evidence-based interventions is imperative to assess long-term health outcomes, inform scale-up of implemented programs, avoid debilitating policy and ethical implications of abandoning funded effective interventions, and maintain community support and trust in research [44]. To maximize the public health impact of evidence-based interventions, it is critical to investigate the factors and processes that influence both implementation and sustainability of these interventions, and the health behaviors and outcomes they produce [44,46]. Adaptability, partnership with organizations delivering the intervention, and commitment from the community have been suggested as key factors in sustaining disease prevention programs [47,48]. The call for sustainable solutions to T2D prevention has yet to be answered [49], with no study to date examining how to sustain evidence-based DPPs in “real-world,” community contexts. Implementing and sustaining evidence-based DPPs has implications for substantially reducing the incidence of T2D in Canada, and beyond, and simultaneously fulfilling an important knowledge gap in implementation science.

Small Steps for Big Changes (SSBC) is an evidence-based DPP, consisting of behavior change techniques for diet and PA modification delivered in six 1-hour sessions. Since development in 2013, SSBC has been systematically scaled up from preliminary efficacy testing in a laboratory setting to real-world effectiveness trials in 1 urban and 5 rural community settings in British Columbia (BC) [37,50-55]. Our research to date has demonstrated that SSBC can be adapted for implementation in community settings, delivered in-person and virtually by “laypersons” with no specialized education or experience [56,57], and results in short- [58], medium- [52,59], and long-term behavior changes [60] (J Bourne et al, unpublished data, 2025) leading to clinically relevant improvements. Most notably, 41% of clients who participate in SSBC no longer have prediabetes (glycated hemoglobin [HbA_1c_] <5.7%; American Diabetes Association criteria) 1 year post program, with a further 16% on the cusp of no longer being prediabetic with HbA_1c_ levels of 5.7% (J Bourne et al, unpublished data, 2025).

**Figure 1:**
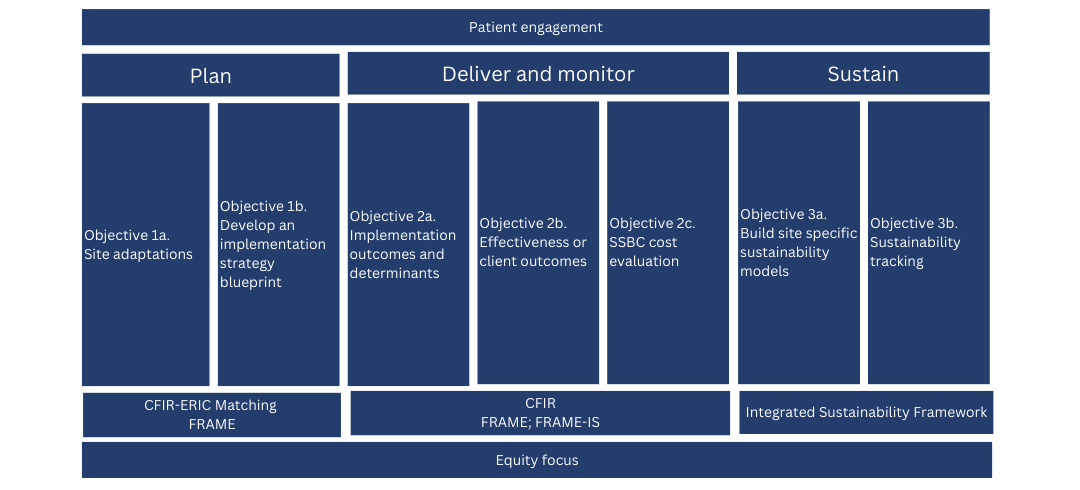
Study objectives. CFIR: Consolidated Framework for Implementation Research; CFIR-ERIC: Consolidated Framework for Implementation Research–Expert Recommendations for Implementing Change; FRAME: Framework for Reporting Adaptations and Modifications-Expanded; FRAME-IS: Framework for Reporting Adaptations and Modifications for Implementation Strategies; SSBC: Small Steps for Big Changes.

Nationwide scale-up of SSBC presents a promising public health solution to equitably reach and improve the health of the growing number of Canadians who are at risk of developing T2D. As such, this project aims to evaluate the implementation, effectiveness, and sustainability of SSBC as it is scaled up nationwide across diverse urban communities in Canada. To address this aim, the project has three objectives (Figure 1): (1) To adapt and implement SSBC in diverse urban cities across Canada; (2) to examine the implementation (determinants, outcomes, and strategies), effectiveness, and cost-effectiveness of SSBC; and (3) determine the sustainability (determinants and outcomes) of delivering SSBC in these contextually varied urban locations.

## Methods

### Study Design and Procedures

**Figure 2:**
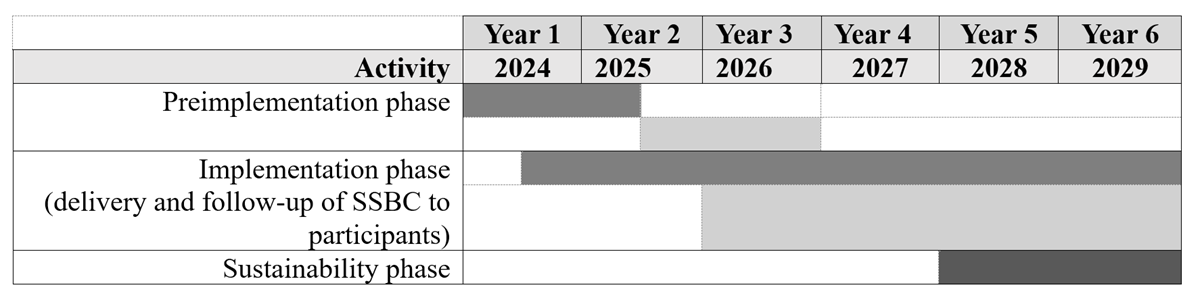
Small Steps for Big Changes delivery sites in Canada. YMCA: Young Men’s Christian Association.

A hybrid type 2 implementation-effectiveness study design [61] using multiple and mixed methods will be used to evaluate the implementation and effectiveness of SSBC over 6 years (2024-2029). Years 1 and 2 will serve as a planning period (preimplementation phase). Specifically, SSBC will be adapted to the site and local contexts, recruitment and referral plans will be developed, and coaches will be trained to deliver SSBC. Years 2-4 will involve the delivery and monitoring of SSBC in diverse urban Young Men’s Christian Association (YMCA) health and fitness facilities (implementation phase). Individuals who enroll in SSBC will be followed for 24 months after completion of the program (until the end of year 6). Data collected during the implementation phase will be used to guide the cocreation of sustainability models with partner organizations for continued delivery of SSBC in each municipality (sustainability phase in years 5 and 6; see Figure 2 for project timeline).

### Intervention

SSBC aims to empower individuals with prediabetes to make long-lasting diet and PA changes. Trained SSBC coaches meet with clients for 6 one-on-one sessions focused on PA, reducing intake of added sugar, improving carbohydrate choices, and preventing relapse, with each session followed by supervised aerobic exercise. Coaches use a motivational interviewing-informed approach to bolster client autonomy. The core components of the SSBC program can be found in Textbox 1, and specific behavior change techniques are published elsewhere [62].

**The core components of Small Steps for Big Changes.**

**Structural elements**

- Six sessions incorporating guidance on diet and physical activity behavior (≈40 minutes) and structured exercise (≈20 minutes).
- Delivered one-on-one.
- Choice-based high-intensity interval training or moderate-intensity continuous training aerobic exercise.
- Coach-supported exercise.
- Counseling style informed by motivational interviewing.

**Context**

- Delivered in the community.
- Deliverable by community lay persons who have received brief virtual coach training.

**Intervention practices (diet and exercise)**

- Brief action planning.
- Relapse prevention planning.
- Self-monitoring.
- Offering education regarding diet (specifically carbohydrate and added sugar content) and physical activity choices.
- Independent practice of goal-oriented diet or physical activity.
- Supporting the client to reflect on behaviors.

### SSBC Coach Training

SSBC is delivered by community-dwelling peers (eg, YMCA staff or volunteers and past clients) without postsecondary education or counseling experience requirements. All individuals who deliver SSBC complete coach training on an online platform, consisting of 7 asynchronous modules, a posttraining knowledge check, and a mock scenario. The mock scenario consists of a coach meeting with an SSBC staff member acting as a standardized client, where the coach must demonstrate correct delivery of intervention content and exercise protocols using a client-centered level of motivational interviewing. The current training was developed with feedback from SSBC coaches [63] and is effective and acceptable [64], with coaches retaining the learnt counseling skills over time (K Cranston et al, unpublished data, 2025).

### Organization Implementation Sites

**Figure 3:**
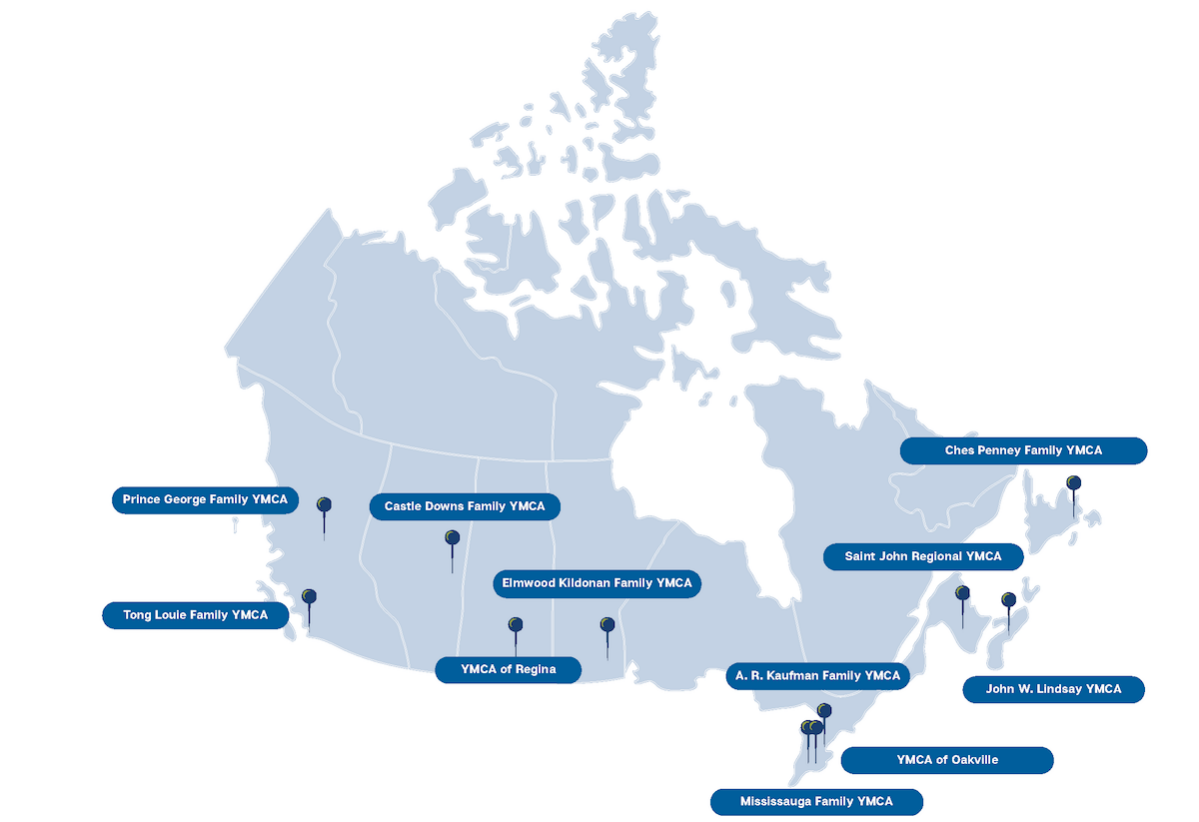
Small Steps for Big Changes Delivery Sites in Canada.

Eleven distinct regional YMCAs across 8 provinces in Canada will participate in this study (see Figure 3 for specific locations). In year 2, SSBC will be delivered in 11 initial implementation sites, one in each distinct YMCA region. Sites were purposively selected to maximize ethnocultural, population density, and socioeconomic diversity, as these contextual factors may influence implementation, effectiveness, and sustainability. Specifically, each site represents a region with high prediabetes prevalence, access to priority populations (operationalized in this project as health equity-owed groups who may face systemic barriers to access, opportunities, and resources to DPPs due to factors such as race, ethnicity, gender, and socioeconomic status; [11-16]), and staffing capacity to deliver SSBC within the organization. Each regional YMCA oversees several health and fitness facility buildings (often in distinct cities) into which SSBC delivery will scale up in years 3 and 4, for a total of 44 Canadian implementation sites.

Each implementation site will select a staff member to act as the site lead for SSBC implementation. The role of each site lead will be to oversee all SSBC coaches and the day-to-day delivery of the program at their site. Senior leadership includes managerial staff (eg, chief executive officer) who are responsible for implementation, site selection, and will help identify site leads, but will not oversee the day-to-day operations of SSBC.

### Data Collection

This study consists of 3 interconnected objectives, highlighted above, that align with the 3 phases of work. Data collection and analysis plans are outlined for each objective below.

### Preimplementation Phase

#### Objective 1a: Adapt SSBC to Each Implementation Site

SSBC has been successfully implemented in 1 urban location in BC [36,50-52]. However, the implementation and sustainability of SSBC in other urban municipalities requires adaptation of these materials and processes. Site-level adaptations will ensure program content is relevant to the local context and delivery is compatible with each site, without altering the core, evidence-based principles of SSBC (Textbox 1). To maximize shared decision-making, an adaptation meeting will be held with each site in the preimplementation phase. This meeting will involve 1 site lead, a minimum of 1 YMCA coach, a regional research coordinator (member of the research team), and the research manager. In addition, regular meetings will be held between the regional research coordinator and the site lead during which program adaptations or the need to make program adaptations will be discussed. All adaptations will be captured using standard templates created by the research team. Site-specific adaptations will be reported and recorded by the research team on an ongoing basis during the implementation phase. Content analysis [65] using the FRAME (Framework for Reporting Adaptations and Modifications-Expanded) [66] will be used to classify adaptations and modifications to SSBC.

#### Objective 1b: Develop an Implementation Strategy Plan and Tracking System

This work will involve developing an implementation strategy plan, or “blueprint,” along with an associated toolkit that formally outlines the strategies for scaling up SSBC across Canada. Implementation strategies are the “methods or techniques used to enhance the adoption, implementation, and sustainability of a program” [43]. The development of this plan will be guided by the identification of barriers and facilitators to SSBC implementation from three sources: (1) findings from the delivery of SSBC at three YMCA facilities in an urban location in BC [50], (2) findings from the delivery of SSBC in five rural locations in BC (K Braaten et al, unpublished data, 2025), and (3) meetings with the research team and YMCA senior leadership in planning for the urban expansion of SSBC. Barriers and facilitators to implementation identified from the 3 sources will be matched to the Consolidated Framework for Implementation Research (CFIR) [67]. The CFIR–Expert Recommendations for Implementing Change matching tool [68] will be used to identify potential implementation strategies that can be used to address each identified factor. Each implementation strategy will be operationalized for the current context, and information on the actor (ie, who delivers the strategy), temporality (ie, when or at what time), and the targeted implementation outcome will be provided such that this information can be easily used by the delivery sites. This process will provide a rigorous and transparent method for linking implementation barriers and facilitators identified across diverse delivery settings to implementation strategies for use in the current project. Use of implementation strategies will be captured through regular meetings between the research team and each delivery site. Content analysis of regular meeting minutes will be conducted using the FRAME-IS (Framework for Reporting Adaptations and Modifications for Implementation Strategies) [69]. In addition, reasons for implementation strategy adaptation, use, or exclusion will be captured. The integrated findings from objectives 1a and 1b will inform the development of each site’s sustainability planning in Objective 3.

### Implementation Phase

#### Objective 2a: Examine the Implementation of SSBC and Associated Implementation Strategies

Implementation will be measured via 4 implementation outcomes at both the level of the intervention and implementation strategies within and between sites. These outcomes include (1) adoption, (2) reach, (3) dose delivered, and (4) fidelity. Table 1 details the implementation outcomes and determinants, how they are operationalized, the source, and the method of data collection. Table 2 provides details of data collection time points for each participant (ie, client, coach, site lead, etc) involved in this study.

**Table 1:** Overview of implementation outcomes and determinants, their operationalization, data source, and measurement tool.

Intervention implementation									Implementation strategies				
Indicators or definition				Data source		Measurement or tool		Indicators or definition			Data source		Measurement or tool
**Implementation outcomes**													
	**Adoption**												
		Number of coaches, proportion of staff who are coaches, and representativeness of coaches	C^a^ and SL^b^		Annual reporting and qualitative interviews		Representativeness of the delivery support team (SL, managers) that use IS^c^			C, SL, and Y-SL^d^		Demographics survey and monthly meetings (SL and RRC^e^)	
		Demographics of coaches and site leads	C and SL		Coach survey		Representativeness of the research team (RRC and DPRG^f^) that uses IS			RRC and DPRG		Demographic survey with the research team	
	**Reach**												
		Number of individuals interested in SSBC^g^ but do not sign up, number of clients enrolled in SSBC, and number that withdraw after enrollment	Client		REDCap^h^ audit of eligibility survey		Number of delivery staff interested, trained, and retained as SSBC coaches			C and SL		Annual report and monthly meetings (SL and RRC); virtual coach training platform tracking	
		Demographic characteristics of clients	Client		Client survey		Recruitment strategies reaching participant			Client		Client eligibility survey	
	**Dose delivered**												
		Number of sessions attended and length of session	C		Session checklists		Number of times IS is used at the delivery site and by the research team			SL, RRC, and DPRG		Monthly meetings (SL and RRC) and the research team IS tracking	
	**Fidelity**												
		Frequency with which participants receive each core component (checklists)	C		Session checklists and audio audits		The extent to which IS was implemented as prescribed in the implementation blueprint			SL		Monthly meetings (SL and RRC)	
		Quality of counseling skills	C		Audio audits of sessions and A-MICA^i^ coded		The extent to which IS was implemented as prescribed in the implementation blueprint			SL		Monthly meetings (SL and RRC)	
**Implementation determinants**													
	**Program cost**												
		Cost per site (trainer costs and delivery time)	SL		Monthly invoices and annual report		Costs incurred from conducting IS			SL, RRC, and DPRG		Annual report and monthly meetings (SL and RRC)	
		Membership referral rates	Client and SL		Client survey and annual report		Costs incurred from conducting IS			SL, RRC, and DPRG		Annual report and monthly meetings (SL and RRC)	
		Retention and conversion (number of YMCA^j^ memberships purchased, SSBC completion)	Client and SL		Client survey and annual report		Costs incurred from conducting IS			SL, RRC, and DPRG		Annual report and monthly meetings (SL and RRC)	
	**Adaptations**												
		Number and type of adaptations made to the SSBC program before delivery	C, SL, and Y-SL		Adaptation workshop minutes and preimplementation tracking sheets		Extent to which IS was adapted, tailored, refined, or reinvented to meet the needs of organizations at scale-up			C, SL, Y-SL, RRC, and DPRG		Monthly meetings (SL and RRC) and interviews with C, SL, Y-SL, RRC, and DPRG	
		Number and type of adaptations made to the SSBC program during delivery	C, SL, and RRC		Monthly meetings (SL and RRC), and C and SL interviews		Extent to which IS was adapted, tailored, refined, or reinvented to meet the needs of organizations at scale-up			C, SL, Y-SL, RRC, and DPRG		Monthly meetings (SL and RRC), and interviews with C, SL, Y-SL, RRC, and DPRG	
		Number and type of adaptations made to sustain SSBC in years 5 and 6	C, SL, Y-SL, and PP^k^		Focus groups and monthly meetings (SL and RRC)		Extent to which IS was adapted, tailored, refined, or reinvented to meet the needs of organizations at scale-up			C, SL, Y-SL, RRC, and DPRG		Monthly meetings (SL and RRC), and interviews with C, SL, Y-SL, RRC, and DPRG	
	**Acceptability**												
		Client satisfaction and acceptability	Client		Client survey (net promoter score, program satisfaction, theoretical framework of acceptability), and interviews		Perceptions among the site lead, coaching staff, or support system that IS are agreeable, palatable, or satisfactory (including SSBC training, program manuals, etc)			C, SL, Y-SL, RRC, and DPRG		Interviews with C, SL, Y-SL, RRC, and DPRG	
		Coach and site lead satisfaction and acceptability	C and SL		C and SL survey (BISS^l^, AIM^m^, IAM^n^, and FIM^o^), and interviews (after 10 clients)		Perceptions among the site lead, coaching staff, or support system that IS are agreeable, palatable, or satisfactory (including SSBC training, program manuals, etc)			C, SL, Y-SL, RRC, and DPRG		Interviews with C, SL, Y-SL, RRC, and DPRG	
	**Context**												
		Aspects of the larger social, political, and economic environment that may influence SSBC implementation	C, SL, Y-SL, and RRC		Monthly meetings (SL and RRC), and interviews with C, SL, and Y-SL. Interview guide based on CFIR^p^ and ISF^q^		Aspects of the larger social, political, and economic environment that may influence the delivery of the implementation strategies			C, SL, Y-SL, RRC, and DPRG		Interviews with C, SL, Y-SL, RRC, and DPRG	
	**Feasibility**												
		Perceptions among the delivery team that SSBC can be successfully used and carried out within their organization	C, SL, Y-SL, and RRC		Monthly meetings (SL and RRC), and interviews with C, SL, and Y-SL. Interview guide based on CFIR and ISF		Perceptions among the delivery team and research team that the implementation strategies can be successfully used or carried out at scale within different organizations or settings			C, SL, Y-SL, RRC, and DPRG		Interviews with C, SL, Y-SL, RRC, and DPRG	
	**Compatibility**												
		Extent to which SSBC fits with the mission, priorities, and values of the delivery site	C, SL, Y-SL, and RRC		Monthly meetings (SL and RRC), and interviews with C, SL, and Y-SL. Interview guide based on CFIR and ISF		Extent to which implementation strategies fit with the mission, priorities, and values of organizations at scale (including SSBC training)			C, SL, Y-SL, and RRC		Interviews: C, SL, Y-SL, and RRC	
	**Culture**												
		The organizations’ norms, values, and basic assumptions around SSBC	C, SL, Y-SL, and RRC		Monthly meetings (SL and RRC), and interviews with C, SL, and Y-SL. Interview guide based on CFIR and ISF		Organizations’ norms, values, and basic assumptions around selected implementation strategies			C, SL, Y-SL, and RRC		Interviews: C, SL, Y-SL, and RRC	
	**Dose (satisfaction)**												
		Delivery team’s satisfaction with SSBC and interactions with the research team	C, SL, Y-SL, and RRC		Monthly meetings (SL and RRC), and interviews with C, SL, and Y-SL. Interview guide based on CFIR and ISF		Delivery team and support systems’ satisfaction with the implementation strategies			C, SL, Y-SL, and RRC		Interviews: C, SL, Y-SL, and RRC	
	**Complexity**												
		Perceptions of the ease or difficulty in implementing SSBC among the delivery team	C, SL, Y-SL, and RRC		Monthly meetings (SL and RRC), and interviews with C, SL, and Y-SL. Interview guide based on CFIR and ISF		Perceptions among the delivery team and support system that implementation strategies are relatively difficult to understand and use, number of different strategies. Related to implementation setting (eg, coach community of practice, SSBC training, and program manual etc)			C, SL, Y-SL, and RRC		Interviews: C, SL, Y-SL, and RRC	
	**Self-efficacy**												
		Coaches’ belief in their ability to execute the course of action to achieve the implementation goals	C, SL, Y-SL, and RRC		Monthly meetings (SL and RRC); interviews with C, SL, and Y-SL. Interview guide based on CFIR and ISF		Support systems’ belief in their ability to execute courses of action to achieve implementation goals			C, SL, Y-SL, and RRC		Interviews: C, SL, Y-SL, and RRC	
	**Organizational readiness**												
		Organizational readiness	C and SL		Survey at baseline using organizational readiness for change [70]		Not applicable			Not applicable		Not applicable	

^a^C: coach.

^b^SL: site lead.

^c^IS: implementation strategies.

^d^Y-SL: Young Men’s Christian Association Senior Leadership.

^e^RRC: Regional Research Coordinator.

^f^DPRG: Diabetes Prevention Research Group.

^g^SSBC: Small Steps for Big Changes.

^h^REDCap: Research Electronic Data Capture (Vanderbilt University).

^i^A-MICA: Abbreviated Motivational Interviewing Competency Assessment.

^j^YMCA: Young Men’s Christian Association.

^k^PP: patient partner.

^l^BISS: Behavioral Interventionist Satisfaction Survey.

^m^AIM: Acceptablity of Intervention Measure.

^n^IAM: Intervention Appropriateness Measure.

^o^FIM: Feasibility of Intervention Measure.

^p^CFIR: Consolidated Framework for Intervention Research.

^q^ISF: Integrated Sustainability Framework.

**Table 2:** Data collection time points.

Who and what			Time point														
			Coach training		Adaptation workshop		Baseline		During delivery		Postprogram completion		12 months into delivery		24 months (end of delivery)		Sustainability (years 3-4)
**SSBC^a^ client**																	
	Consent					✓											
	Intake survey					✓											
	Questionnaires and in-person measures					✓				✓		✓		✓			
	Interviews (2-3 clients per site)									✓^b^							
	Site focus group^c^													✓		✓ (x2)	
**SSBC coach**																	
	Coach training and demographics	✓															
	Consent					✓											
	Adaptation workshop			✓													
	Survey					✓ (ORQ^d^)		✓ (BISS^e^, AIM^f^, IAM^g^, FIM^h^; at 5, 10, and 20 clients if applicable)									
	Delivery checklists or audio audit							✓									
	Interviews (2 coaches per site)							✓^i^ (10 clients)									
	Site focus groups													✓		✓ (x2)	
**Delivery site lead**																	
	Consent					✓											
	Coach training and demographics	✓															
	Adaptation workshop			✓													
	Survey					✓ (ORQ)						✓ (AIM, IAM, and FIM)					
	Monthly meetings					✓		✓		✓		✓		✓			
	Interviews											✓		✓			
	Site focus groups													✓		✓ (x2)	
**Delivery site senior leadership**																	
	Consent and demographics											✓					
	Meeting minutes																
	Interviews											✓		✓			
	Site focus groups													✓		✓ (x2)	
	Municipality partners^j^: focus group													✓			
	Regional research coordinator: interview													✓			
	Diabetes prevention research group project manager: interview													✓			

^a^SSBC: Small Steps for Big Changes.

^b^These interviews will occur 2-3 months after the client has completed Small Steps for Big Changes.

^c^Site focus groups focused on creating sustainability models if deemed appropriate and identifying implementation strategies used.

^d^ORQ: Organizational Readiness Questionnaire.

^e^BISS: Behavioral Interventionist Satisfaction Survey.

^f^AIM: Acceptability of Intervention Measure.

^g^IAM: Intervention Appropriateness Measure.

^h^FIM: Feasibility of Intervention Measure.

^i^Coaches must have had 10 clients complete the Small Steps for Big Changes before being interviewed. Note that quantitative measures at 10 clients are to be completed ahead of the interview.

^j^Municipality partners include patient partners, Small Steps for Big Changes alumni, and persons with lived experience.

In addition, the multilevel factors posited to influence intervention implementation and use of implementation strategies (ie, the implementation determinants) will be examined across multiple settings and contexts. This information will be captured qualitatively through monthly meetings between the research team and delivery sites and through one-on-one interviews with 2 coaches at each site, site leads (after 1 year of delivery), and senior leadership at each site (after 2 years of delivery). Interview guides will be guided by the CFIR [67] to understand the contextual factors, organizational factors, processes factors, implementer characteristics, and intervention characteristics that impact implementation.

Quantitatively, organizational readiness will be assessed at baseline [70] through surveys with site leads and coaches. Implementer satisfaction with the program will be captured using the Behavioral Interventionist Satisfaction Survey [71] and the Intervention Acceptability, Appropriateness, and Feasibility [72] measures during the delivery process. Client receptivity and satisfaction will be assessed quantitatively using the 8-item Theoretical Framework for Acceptability [73], a 7-item overall program satisfaction measure, and the 1-item net promoter score [74]. In addition, client interviews will be conducted with a purposive subsample (2-3 clients per site) post-SSBC completion to assess client satisfaction and acceptability. Program costs will be captured through monthly invoices and annual reports from sites.

Quantitative data from adoption, reach, dose, fidelity, program cost, and client and coach receptivity measures will be analyzed descriptively. Client demographics will be used to assess representativeness relative to people with prediabetes across Canada. SSBC program sessions will be audio-recorded using tablets with data saved on the University of British Columbia’s secure data research network in real-time; 2 program sessions per year from each coach will be randomly sampled and coded using an abbreviated version of the validated Motivational Interviewing Competency Assessment [75]. Motivational Interviewing Competency Assessment scores will be used to assess coach counseling fidelity, as done previously [57]. All implementation strategies used will be identified and classified according to definitions from the Expert Recommendations for Implementing Change project [76].

Qualitative data from interviews will be audio-recorded, transcribed verbatim, and analyzed using coding reliability thematic analysis [77]. This method aligns with the pragmatic research objectives for these interviews to assess satisfaction and acceptability (clients) and barriers and facilitators of implementation (delivery team), thereby supporting considerations of meaningful and methodological coherence [78]. Interviews will be deductively coded by 2 independent coders to increase the credibility and transferability of the findings with the aim of arriving at one objective truth [78]. Additionally, selecting an aim that will improve the practice of implementation efforts and offer significant contributions to the field further considers rigor and quality in qualitative inquiry [78].

#### Objective 2b: Examine the Effectiveness of SSBC

All clients will complete in-person measurements (weight and waist circumference) at baseline, 12-, and 24-month post program. At locations with an appropriate space (ie, walking track), cardiorespiratory fitness will also be assessed via a 6-minute walk test [79]. In addition, all clients will complete surveys at baseline, session 6, 12, and 24 months post program via REDCap (Research Electronic Data Capture). Surveys will assess self-reported T2D status (12- and 24-months only [80,81]), moderate-to-vigorous PA, dietary intake, and health-related quality of life (EQ-5D-5L) [82]. Table 3 outlines the measures that will be used in the evaluation of SSBC effectiveness. Using an equity-focused lens, a prospective cohort subsample of clients (n=1285) will be followed across 24 months to evaluate the differential impact of SSBC on T2D status, assessed by change in HbA_1c_, in addition to self-reported T2D status. All effectiveness outcomes will be analyzed using descriptive statistics. Hierarchical multivariable regression models will be used to assess the impact of SSBC on effectiveness outcomes, adjusting for potential confounders and accounting for within-subject and within-site clustering. Covariates (ie, sex, gender, and ethnicity) will be included to adjust for within-subject and within-site correlations. A sensitivity analysis using missing data imputation will be conducted to evaluate the potential impact of nonadherence on study outcomes. Data will be disaggregated to examine differences in effectiveness by ethnicity, socioeconomic status, municipality, sex, and gender within and between sites. For the subsample, a multilevel binary logistic regression will be used to examine differences in sociodemographic factors between those who do and do not experience a clinically significant reduction in HbA_1c_ at 24 months.

**Table 3:** Overview of effectiveness measures and associated time points.

		Eligibility	Baseline measurement	Postprogram measurement	Follow-up measurements	
Measure		Online intake survey	Baseline on session 1	After session 6	12-month follow-up	24-month follow-up
Age		✓				
Current T2D^a^ diagnosis		✓				
Prediabetes diagnosis or ADA^b^ diabetes risk score		✓				
**T2D status**						
	1-item self-report T2D status (in questionnaire)		✓		✓	✓
	Laboratory-based HbA_1c_^c^ (subsample)		✓		✓	✓
**Client questionnaire**						
	Demographic characteristics		✓			
	GLTEQ^d^ [83]		✓	✓	✓	✓
	PAV^e^ [84]		✓	✓	✓	✓
	4-item 24-hour movement measure (adapted for SSBC^f^ urban scale up in line with Canadian 24-hour movement guidelines [85])		✓	✓	✓	✓
	Brief Food Frequency Measure [86]		✓	✓	✓	✓
	Self-compassion [87]		✓			
	EuroQol-5D-5 level HRQOL^g^, Weighted Health State Utility Value [82]		✓	✓	✓	✓
	HRU^h^ [88]		✓		✓	✓
	General program feedback (7 items created for SSBC: scored on a scale of 1=extremely dissatisfied to 7=extremely satisfied)			✓		
	Program acceptability (8-item theoretical framework of acceptability [73])			✓		
	Net promoter score (1-item)			✓		
	Membership (1-item)			✓		
	Referral (3-items)			✓		
**In-person measurements**						
	Height (m)		✓			
	Weight (lbs)		✓	✓	✓	✓
	Waist circumference (cm)		✓	✓	✓	✓
	Resting heart rate (BPM)		✓	✓	✓	✓
	CRF^i^ (6MWT^j^) [89]		✓	✓	✓	✓

^a^T2D: type 2 diabetes.

^b^ADA: American Diabetes Association.

^c^HbA_1c_: glycated hemoglobin.

^d^GLTEQ: Godin Leisure Time Exercise Questionnaire.

^e^PAV: Physical Activity Vital Sign.

^f^SSBC: Small Steps for Big Changes.

^g^HRQOL: Health Related Quality of Life.

^h^HRU: Health Resource Utilization Questionnaire.

^i^CRF: cardiorespiratory fitness.

^j^6MWT: 6-minute walk test.

#### Objective 2c: Examine the Cost-Effectiveness of SSBC

The cost-effectiveness of SSBC will be determined using organizational data collected in objective 2a and client-level costs. Health-related client-level costs will be detailed using the Health Resource Utilization Questionnaire [88] delivered in a survey. The Health Resource Utilization Questionnaire asks for specific details regarding health professional visits, admissions to the hospital, laboratory tests or investigations, and use of medications. On a per-client basis, costs will be assigned to health care resource use using a fully allocated hospital cost model (for in-patient costs) and provincial guides to medical fees (for outpatient costs) every 6 months to account for potential fluctuations in medical costs. The 5 dimensions of EQ-5D-5L will be converted into a weighted health state utility value that will provide weightings for quality-adjusted life years (QALYs) and Canadian conversion tariffs for transforming health state profiles into utility scores [90]. QALYs are calculated based on the quality of life of a person (measured using health state utility values estimated from the EQ-5D-5L) in a given health state and the time spent in that health state. Incremental cost per client and effects generated by SSBC will be assessed using a Canadian health care system perspective. The outcome of cost-effectiveness analysis is the incremental cost-effectiveness ratio, where incremental cost-effectiveness ratio=Δ Cost/Δ HbA_1c_ and is the incremental cost of SSBC delivery per client. The outcome of our cost-utility analysis is the incremental cost-utility ratio, where incremental cost-utility ratio=Δ Cost/Δ QALY [91].

### Sustainability Phase

#### Objective 3a: Cocreate Sustainability Models

In collaboration with the partnering YMCAs and affiliated municipality partners, the research team will engage in a process of codeveloping a feasible and reciprocally beneficial model of sustaining SSBC within each organization. Data from objectives 1 and 2 will be used to generate reports for each YMCA to directly inform this phase. Next, focus groups will be held at each site. Guided by the Integrated Sustainability Framework (ISF) [44], municipality-level models will establish which program components of SSBC will be continued (if at all), and at what intensity. Focus groups will also elucidate what factors result in the sustainment of evidence-based interventions in community settings. The outcome of this objective will be the development of a logic model of sustainment satisfactory to all parties.

#### Objective 3b: Examine SSBC Sustainability

Sustainability of SSBC will be examined at the individual client level (the number of clients who enroll and complete SSBC in the sustainability phase), individual coach level (the number of coaches that continue to deliver SSBC in the sustainability phase), and the site level (the number of sites that continue SSBC delivery in the sustainability phase; see Table 4). Potential contextual factors that influence the sustainability of SSBC will be explored through interviews with site leads and senior leadership 1 year into the sustainability phase of the project (2028) and at the end of the sustainability phase (2029). Interview guides will be developed using the ISF [44]. In addition, regional research coordinators will document any contextual factors impacting sustainability during regular meetings with site leads. All sustainability determinants will be deductively coded to the categories represented in the ISF (outer context, organizational context, processes, intervention characteristics, and implementer characteristics) by 2 independent coders. Implementation strategies used during this sustainability phase will be documented and analyzed as per the same processes used for implementation.

**Table 4:** Overview of sustainability outcomes and determinants, their operationalization, data sources, and measurement tools. The outcome is sustainability.

Indicators or definition	Data source	Measurement tool and time point
Site level: number of sites continuing delivery, intention to deliver the program, actual delivery of the program, and attempts to seek funds to run the program if appropriate	SL^a^, Y-SL^b^, and RRC^c^	Interviews with SL and Y-SL, focus groups, annual monitoring, and monthly meetings with RRC
Staff level: number of coaches continuing to deliver the program	SL and RRC	Site monthly meetings during the sustainability phase and annual reporting
Individual level: number of clients who participated in the presustainability phase and number who participated in the sustainability phase, if applicable	SL and RRC	Site monthly meetings with RRC and REDCap^d^
Sustainability determinants: contextual factors as assessed through the ISF at 24 months into implementation, after 1 year of sustainability, and end of sustainability	C^e^, SL, Y-SL, MP^f^, PP^g^, and RRC	Interviews, focus groups, and site monthly meetings with RRC
Implementation strategies: strategies continued, discontinued, and reasons	C, SL, Y-SL, MP, PP, and RRC	Interviews, focus groups, and site monthly meetings with RRC

^a^SL: site lead.

^b^Y-SL: Young Men’s Christian Association senior leadership.

^c^RRC: Regional Research Coordinator.

^d^REDCap: Research Electronic Data Capture.

^e^C: coach.

^f^MP: municipality partners.

^g^PP: patient partner.

#### Recruitment and Consent

##### SSBC Clients

Recruitment strategies will vary across delivery sites, based on the local connections previously or newly established by the research team and delivery team. A series of generic recruitment materials (eg, posters, pamphlets, or social media posts) and potential recruitment pathways (eg, health assessment days and physician referrals) have been generated by the research team and will be shared with all delivery sites. These recruitment strategies will be included in the implementation strategies’ blueprint and toolkit. Each site will tailor their recruitment plan to the local contextual needs with support from the research team.

Interested or referred individuals will complete an eligibility survey to determine their eligibility for SSBC. Individuals are eligible for SSBC if they are 18 years of age or older, able to read and speak English, and meet one of the following criteria: (1) have been diagnosed with prediabetes by a physician, (2) have an HbA_1c_ of 5.7%-6.4%, (3) have an American Diabetes Association risk assessment score indicating high risk (≥5), or (4) have achieved T2D remission (have an HbA_1c_ of <6.5%) without any diabetes-related medications for a minimum of 3 months. Eligible individuals will be directed to an online consent form. Following completion, individuals will be scheduled for their first SSBC session at their local YMCA implementation site.

Individuals who express an interest in the substudy will be given a blood requisition form for an HbA_1c_ measurement from a partnering local blood requisition laboratory. The substudy will aim to oversample individuals identifying as visible minority to ensure that priority populations are represented in line with the most recent Canadian Government statistics (based and derived directly from the concept of visible minority in the census [31.3% of the Canadian population identify as being from a racialized group, with 25.2% as visible minority and 6.1% from Indigenous ancestry]). The substudy will also oversample individuals identifying as male, who are at greater risk for having prediabetes and developing T2D [6], yet are disproportionately represented in DPPs [92]. Demographic information of clients enrolling in the substudy will be assessed every 6 months to determine the proportion of individuals self-identifying as visible minorities and males. Purposive sampling may be used during the substudy if the demographic data of subsample clients demonstrates over- or underrepresentation.

##### SSBC Delivery Team

YMCA staff from the delivery sites will be invited to participate in this study to provide feedback on adaptation, implementation processes, and factors influencing implementation and sustainability of SSBC at their site. Site leads and coaches will be identified by senior leadership at each YMCA facility. After completing coach training, site leads and coaches will receive a consent form to participate in surveys, interviews, and focus groups. Senior leadership will receive a consent form 12 months into the program delivery, inviting them to participate in interviews and focus groups.

##### Project Governance

**Figure 4:**
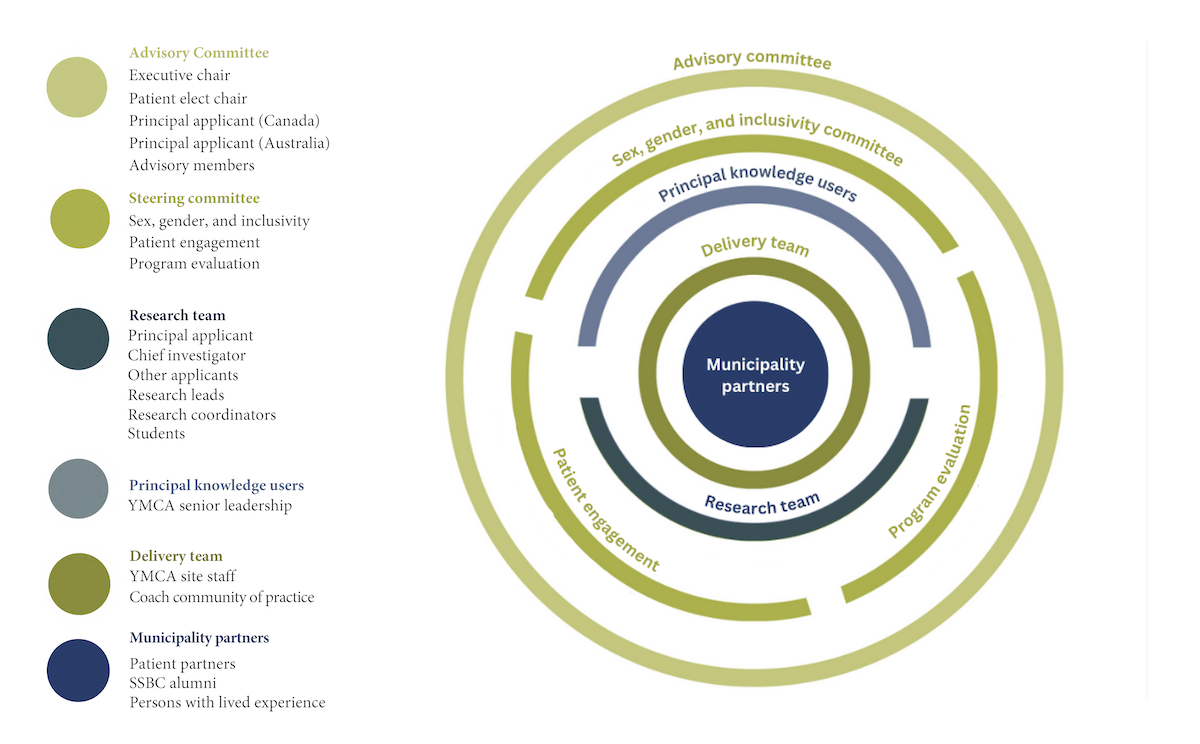
Project governance model. SSBC: Small Steps for Big Changes; YMCA: Young Men’s Christian Association.

A holacratic approach to organizational operations was codeveloped with delivery sites to enhance conflict resolution and achieve project objectives. Specifically, each team member is considered integral and acknowledges their interconnected role in multiple project processes and outcomes (Figure 4). Municipality partners (made up of patient partners, SSBC alumni, and persons with lived experience) are central to advising the project. The delivery team—the community-dwelling staff who deliver SSBC and liaise directly with researchers, senior YMCA leadership, and municipality partners—will provide ongoing feedback on referral and recruitment strategies, SSBC implementation and adaptations, and factors influencing organizational and staff readiness. YMCA senior management at each site oversees the delivery team and reports to the research team. Three committees will provide feedback and guidance on methods used, analyses performed, outputs reported, and the sustainability plan based on their expertise and experiences for sex, gender, and inclusivity; program evaluation; and the diabetes prevention engagement committee. Finally, an advisory committee will act as an external committee distinct from the project team. This committee will ensure that safe and ethical principles are applied throughout the project, project milestones are met promptly, and all decisions are made with clients and delivery organizations in mind.

### Ethical Considerations

This study has been approved by the University of British Columbia Clinical Research Ethics Board (H23-01930) and other university and provincial ethics boards (Brock University Health Science Research Ethics Board [REB-23-157] and Newfoundland and Labrador Health Research Ethics Board [HREB 20250577]). All activities will comply with the Tri-Council Policy Statement: Ethical Conduct of Research Involving Humans. Written informed consent will be obtained from all participants. Privacy and confidentiality of participant data or identity will be maintained. Consent forms will be stored separately from deidentified data. Pseudonyms will be used in interview transcripts and any reporting of the results. Participants will be compensated for their participation. Individuals who participate in the program will receive a 1-month free membership to the facility they attend. All client participants will receive a CAD $20 gift card for each of the follow-up appointments that they attend (CAD $40 total), as well as a CAD $30 gift card for each time they get their bloodwork done if they are part of the subsample (CAD $90 total). All participants who participate in an interview or focus group will receive a CAD $30 gift card. This study has been registered at ClinicalTrials.gov. The Standards for Reporting Implementation Studies checklist [93] was used to guide the reporting of this protocol paper and can be found in Multimedia Appendix 1.

## Results

Program implementation within each region is occurring in a phased approach, with partners beginning program delivery in 1 site (2024-2025) before expanding to any additional locations (2025-2027). Program enrollment occurs continuously during the implementation phase across all sites. From May 2024 to November 2025, a total of 13 delivery sites began program delivery (1 regional YMCA decided to launch in 3 sites at once), 722 participants have enrolled in the program, and 406 have begun the program (153/342, 44.7% non-Western or Eastern European, 17/342, 5% of whom report identifying as Indigenous; 80/345, 23.2% men or 82/347, 23.6% male). Further, 334 participants have expressed interest in the substudy, of which 89 have had their first blood draw (19/89, 21.8% identify as a visible minority; 4/89, 5.3% indigenous; 16/82, 19.5% men or 16/81, 19.8% male). An additional 13 sites have confirmed they will launch the program in 2026.

## Discussion

### Principal Findings

This study will provide evidence that SSBC can be scaled up nationwide across community settings to equitably and effectively reduce population-level risk of T2D. Nationwide uptake of DPPs has been previously hindered by the absence of evidence-informed implementation plans tailored to local contexts [38]. This project will identify which implementation strategies work best for whom, where, and how, deepening our understanding of how to translate SSBC to multiple contexts. We move beyond a one-size-fits-all strategy to apply evidence-based implementation principles that will result in equitable program implementation for diverse and priority populations across Canada. This protocol paper documents our key objectives and associated methods for other implementation scientists outside of the realm of T2D prevention to consider when planning to implement evidence-based interventions at scale.

### Strengths and Limitations

Sustainable DPP scale-up is a necessary condition for improved population health outcomes. This is the first study to examine how to sustain an evidence-based DPP in real-world community contexts. Unlike other DPPs that have been delivered at a national scale (eg, US National DPP [94] and English National DPP [95]), SSBC was designed to include characteristics that are key for achieving sustainability—for example, being brief, adaptable, community-based, and delivered by laypersons with no specialized training. By exploring the underlying factors underpinning the dynamic processes of implementation and sustainability when delivered at a broad scale, and engaging early with municipality partners to develop and prioritize sustainability planning to overcome identified barriers, this research will discern best practice implementation determinants, outcomes, and strategies critical for sustaining DPP implementation across Canada and beyond.

Implementation science is critical in improving health equity by ensuring health programs are accessible and effective to those most in need [40,41]. Of the participants that have enrolled thus far, we are on track for reaching an ethnically representative sample of the Canadian population, which will allow us to understand if SSBC is effective for visible minorities; however, individuals self-identifying as men and male continue to be underrepresented in SSBC participation. Establishing buy-in from health care professionals in clinical settings may help us to reach this demographic, but developing partnerships with this sector has proven challenging. Extending diabetes prevention to community settings is key to advancing health equity; however, staff shortages and turnover are ongoing challenges faced when working with community partners.

### Dissemination Plan

End-of-project and integrated knowledge translation activities are planned and designed around fundamental knowledge translation principles [96-98]. To ensure broad reach, we will share our knowledge with multiple audiences using multiple formats (see Table 5 for a selected list of knowledge mobilization activities).

**Table 5:** Select knowledge mobilization activities for the national scale-up of Small Steps for Big Changes.

Key audiences and sectors for KT^a^	Selected sample tactics or knowledge products (outputs to audiences)	Sample tactics for knowledge input from audiences to the partnership
Delivery Team (YMCA^b^ staff, senior leadership), their organizational stakeholders (donors, board of directors, and government) and the health and recreation sector	Post resources and peer-delivered information on the project’s website and social media accounts Papers in YMCA and stakeholder magazines, newsletters, etc Mass media press releases Flow research and knowledge products through YMCA + stakeholders’ networks (eg, present at AGMsc, in annual reports, etc) Publish papers in open-access journals Publish papers in trade journals Develop and disseminate evidence-based guides, toolkits, videos, and decision aids	Advisory committee and committee meetings Coach community of practice (ongoing) Site lead community of practice Knowledge-sharing meetings (2/year) Train the trainer website (ongoing) Board of directors meetings
Organizations providing services to diverse communities at T2D^d^ risk (eg, immigration services, indigenous friendship centers, or food banks)	Disseminate 1-page project briefs and summary reports Host webinars and post resources and information on the project and partners’ websites and social media accounts Present at organizations’ AGMs and other events Publication in open-access journals Mass media press releases	Advisory committee and committee discussions with organizations Municipality partners committed to supporting SSBCe
Policy makers (municipality stakeholders, First Nation, and other health authorities)	1-page policy briefs that will be brought forward at our scheduled meetings with Government and Health Authority Partners Reports for government ministries	Meetings with municipality and health partners: when and how to influence policy decisions re: T2D prevention and Bill C-237
Academics and Diabetes Care Practitioners	Peer-reviewed journals Present at academic conferences	Engagement with National Diabetes Framework plan and funding calls Identify relevant and impactful journals and conferences

^a^KT: knowledge translation.

^b^YMCA: Young Men’s Christian Association.

^c^AGM: Annual General Meeting.

^d^T2D: type 2 diabetes.

^e^SSBC: Small Steps for Big Changes.

## Appendix

Multimedia Appendix 1 Peer review report from Healthy Cities Implementation Science (HCIS) Team Grants Review Committee, Canadian Institutes for Health Research (CIHR).

## Abbreviations

BC: British Columbia
CFIR: Consolidated Framework for Implementation Research
DPP: diabetes prevention program
FRAME: Framework for Reporting Adaptations and Modifications-Expanded
FRAME-IS: Framework for Reporting Adaptations and Modifications for Implementation Strategies
HbA1c: glycated hemoglobin
ISF: Integrated Sustainability Framework
PA: physical activity
QALY: quality-adjusted life year
REDCap: Research Electronic Data Capture
SSBC: Small Steps for Big Changes
T2D: type 2 diabetes
YMCA: Young Men’s Christian Association

## Data Availability

The future datasets generated or analyzed during this study will be made available from the corresponding author on reasonable request.

## References

[1] GBD 2021 Diabetes Collaborators (2023). Global, regional, and national burden of diabetes from 1990 to 2021, with projections of prevalence to 2050: a systematic analysis for the Global Burden of Disease study 2021. Lancet.

[2] Magliano DJ, Boyko EJ (2021). IDF Diabetes Atlas 10th edition scientific committee. IDF Diabetes Atlas.

[3] Haw JS, Galaviz KI, Straus AN, Kowalski AJ, Magee MJ, Weber MB, Wei J, Narayan KMV, Ali MK (2017). Long-term sustainability of diabetes prevention approaches: a systematic review and meta-analysis of randomized clinical trials. JAMA Intern Med.

[4] Schellenberg ES, Dryden DM, Vandermeer B, Ha C, Korownyk C (2013). Lifestyle interventions for patients with and at risk for type 2 diabetes: a systematic review and meta-analysis. Ann Intern Med.

[5] Uusitupa M, Khan TA, Viguiliouk E, Kahleova H, Rivellese AA, Hermansen K, Pfeiffer A, Thanopoulou A, Salas-Salvadó J, Schwab U, Sievenpiper JL (2019). Prevention of type 2 diabetes by lifestyle changes: a systematic review and meta-analysis. Nutrients.

[6] (2024). Diabetes in Canada: estimated prevalence and cost of diabetes. Diabetes Canada.

[7] (2018). Diabetes 360: a framework for a diabetes strategy for Canada. Diabetes Canada.

[8] Tabák AG, Herder C, Rathmann W, Brunner EJ, Kivimäki M (2012). Prediabetes: a high-risk state for diabetes development. Lancet.

[9] (2018). Key health inequalities in Canada: a national portrait - executive summary. Public Health Agency of Canada.

[10] Fazli GS, Moineddin R, Bierman AS, Booth GL (2019). Ethnic differences in prediabetes incidence among immigrants to Canada: a population-based cohort study. BMC Med.

[11] D'Silva C, Hafleen N, Mansfield E, Martel S, Fierheller D, Banerjee A, Malhotra G, Mutta B, Dhillon P, Hasan Z, Parikh A, Nooraie RY, Chaze F, Zenlea I (2022). Service provider perspectives on exploring social determinants of health impacting type 2 diabetes management for South Asian adults in peel region, Canada. Can J Diabetes.

[12] Scheim AI, Coleman T, Lachowsky N, Bauer GR (2021). Health care access among transgender and nonbinary people in Canada, 2019: a cross-sectional survey. CMAJ Open.

[13] Tami A, Ferguson T, Bauer GR, Scheim AI (2022). Avoidance of primary healthcare among transgender and non-binary people in Canada during the COVID-19 pandemic. Prev Med Rep.

[14] Fante-Coleman T, Wilson CL, Cameron R, Coleman T, Travers R (2022). 'Getting shut down and shut out': exploring ACB patient perceptions on healthcare access at the physician-patient level in Canada. Int J Qual Stud Health Well-Being.

[15] Socías ME, Koehoorn M, Shoveller J (2016). Gender inequalities in access to health care among adults living in British Columbia, Canada. Womens Health Issues.

[16] (2020). In plain sight: addressing indigenous-specific racisim and discrimination in B.C. health care. BC Ministry of Health.

[17] (2022). Framework for diabetes in Canada. Canada.

[18] Knowler WC, Barrett-Connor E, Fowler SE (2002). Reduction in the incidence of type 2 diabetes with lifestyle intervention or metformin. N Engl J Med.

[19] Pan XR, Li GW, Hu YH, Wang JX, Yang W, An Z, Hu Z X, Lin J, Xiao JZ, Cao HB, Liu PA, Jiang XG, Jiang YY, Wang JP, Zheng H, Zhang H, Bennett PH, Howard BV (1997). Effects of diet and exercise in preventing NIDDM in people with impaired glucose tolerance: the Da Qing IGT and diabetes study. Diabetes Care.

[20] Lindström J, Louheranta A, Mannelin M, Rastas M, Salminen V, Eriksson J, Uusitupa M, Tuomilehto J, Finnish Diabetes Prevention Study Group (2003). The Finnish Diabetes Prevention Study (DPS): lifestyle intervention and 3-year results on diet and physical activity. Diabetes Care.

[21] Chambers DA, Glasgow RE, Stange KC (2013). The dynamic sustainability framework: addressing the paradox of sustainment amid ongoing change. Implement Sci.

[22] Lane C, McCrabb S, Nathan N, Naylor PJ, Bauman A, Milat A, Lum M, Sutherland R, Byaruhanga J, Wolfenden L (2021). How effective are physical activity interventions when they are scaled-up: a systematic review. Int J Behav Nutr Phys Act.

[23] Ariel-Donges AH, Gordon EL, Dixon BN, Eastman AJ, Bauman V, Ross KM, Perri MG (2020). Rural/urban disparities in access to the National Diabetes Prevention Program. Transl Behav Med.

[24] Venkataramani M, Pollack CE, Yeh HC, Maruthur NM (2019). Prevalence and correlates of diabetes prevention program referral and participation. Am J Prev Med.

[25] French DP, Hawkes RE, Bower P, Cameron E (2021). Is the NHS diabetes prevention programme intervention delivered as planned? An observational study of fidelity of intervention delivery. Ann Behav Med.

[26] Hawkes RE, Cameron E, Miles LM, French DP (2021). The fidelity of training in behaviour change techniques to intervention design in a National Diabetes Prevention Programme. Int J Behav Med.

[27] Hawkes RE, Miles LM, Bower P, Cotterill S, French DP (2022). Assessing and ensuring fidelity of the nationally implemented English NHS diabetes prevention programme: lessons learned for the implementation of large-scale behaviour change programmes. Health Psychol Behav Med.

[28] Azar KMJ, Nasrallah C, Szwerinski NK, Petersen JJ, Halley MC, Greenwood D, Romanelli RJ (2019). Implementation of a group-based diabetes prevention program within a healthcare delivery system. BMC Health Serv Res.

[29] Brunisholz KD, Kim J, Savitz LA, Hashibe M, Gren LH, Hamilton S, Huynh K, Joy EA (2017). A formative evaluation of a diabetes prevention program using the RE-AIM framework in a learning health care system, Utah, 2013-2015. Prev Chronic Dis.

[30] Whittemore R (2011). A systematic review of the translational research on the diabetes prevention program. Transl Behav Med.

[31] Venditti EM, Wylie-Rosett J, Delahanty LM, Mele L, Hoskin MA, Edelstein SL, Diabetes Prevention Program Research Group (2014). Short and long-term lifestyle coaching approaches used to address diverse participant barriers to weight loss and physical activity adherence. Int J Behav Nutr Phys Act.

[32] McCurley JL, Gutierrez AP, Gallo LC (2017). Diabetes prevention in U.S. Hispanic adults: a systematic review of culturally tailored interventions. Am J Prev Med.

[33] Ritchie ND, Christoe-Frazier L, McFann KK, Havranek EP, Pereira RI (2018). Effect of the National Diabetes Prevention Program on weight loss for English- and Spanish-speaking latinos. Am J Health Promot.

[34] Ritchie ND, Phimphasone-Brady P, Sauder KA, Amura CR (2021). Perceived barriers and potential solutions to engagement in the National Diabetes Prevention Program. ADCES Pract.

[35] Garst J, L'Heveder R, Siminerio LM, Motala AA, Gabbay RA, Chaney D, Cavan D (2017). Sustaining diabetes prevention and care interventions: a multiple case study of translational research projects. Diabetes Res Clin Pract.

[36] Bean C, Sewell K, Jung ME (2020). A winning combination: collaborating with stakeholders throughout the process of planning and implementing a type 2 diabetes prevention programme in the community. Health Soc Care Community.

[37] Wurz A, Bean C, Shaikh M, Culos-Reed SN, Jung ME (2022). From laboratory to community: three examples of moving evidence-based physical activity into practice in Canada. Health Soc Care Community.

[38] Gruss SM, Nhim K, Gregg E, Bell M, Luman E, Albright A (2019). Public health approaches to type 2 diabetes prevention: the US national diabetes prevention program and beyond. Curr Diab Rep.

[39] Aziz Z, Mathews E, Absetz P, Sathish T, Oldroyd J, Balachandran S, Shetty SS, Thankappan KR, Oldenburg B (2018). A group-based lifestyle intervention for diabetes prevention in low- and middle-income country: implementation evaluation of the Kerala Diabetes Prevention Program. Implement Sci.

[40] Brownson RC, Kumanyika SK, Kreuter MW, Haire-Joshu D (2021). Implementation science should give higher priority to health equity. Implement Sci.

[41] Baumann AA, Cabassa LJ (2020). Reframing implementation science to address inequities in healthcare delivery. BMC Health Serv Res.

[42] Durlak JA, DuPre EP (2008). Implementation matters: a review of research on the influence of implementation on program outcomes and the factors affecting implementation. Am J Community Psychol.

[43] Proctor EN, Powell BJ, McMillen JC (2013). Implementation strategies: recommendations for specifying and reporting. Implement Sci.

[44] Shelton RC, Cooper BR, Stirman SW (2018). The sustainability of evidence-based interventions and practices in public health and health care. Annu Rev Public Health.

[45] Proctor E, Luke D, Calhoun A, McMillen C, Brownson R, McCrary S, Padek M (2015). Sustainability of evidence-based healthcare: research agenda, methodological advances, and infrastructure support. Implement Sci.

[46] Glasgow RE, Chambers D (2012). Developing robust, sustainable, implementation systems using rigorous, rapid and relevant science. Clin Transl Sci.

[47] Scheirer MA, Dearing JW (2011). An agenda for research on the sustainability of public health programs. Am J Public Health.

[48] Shediac-Rizkallah MC, Bone LR (1998). Planning for the sustainability of community-based health programs: conceptual frameworks and future directions for research, practice and policy. Health Educ Res.

[49] Wareham NJ, Herman WH (2016). The clinical and public health challenges of diabetes prevention: a search for sustainable solutions. PLoS Med.

[50] Dineen TE, Bean C, Jung ME (2022). Implementation of a diabetes prevention program within two community sites: a qualitative assessment. Implement Sci Commun.

[51] Dineen TE, Banser T, Bean C, Jung ME (2021). Fitness facility staff demonstrate high fidelity when implementing an evidence-based diabetes prevention program. Transl Behav Med.

[52] Bean C, Dineen T, Locke SR, Bouvier B, Jung ME (2021). An evaluation of the reach and effectiveness of a diabetes prevention behaviour change program situated in a community site. Can J Diabetes.

[53] Dineen TE, Bean C, Jung ME (2024). Successes and challenges from a motivational interviewing-informed diabetes prevention program situated in the community. Health Promot Pract.

[54] Cranston KD, MacPherson MM, Sim JA, Jung ME (2023). Small steps towards an inclusive diabetes prevention program: how small steps for big changes is improving program equity and inclusion. Community Health Equity Res Policy.

[55] MacPherson M, Merry K, Locke S, Jung M (2022). Developing mobile health interventions with implementation in mind: application of the multiphase optimization strategy (MOST) preparation phase to diabetes prevention programming. JMIR Form Res.

[56] McGavock J, Chauhan BF, Rabbani R, Dias S, Klaprat N, Boissoneault S, Lys J, Wierzbowski AK, Sakib MN, Zarychanski R, Abou-Setta AM (2020). Layperson-led vs professional-led behavioral interventions for weight loss in pediatric obesity: a systematic review and meta-analysis. JAMA Netw Open.

[57] Cranston K, Ivanova E, Davis C, Jung M Fidelity and maintenance of motivational interviewing skills in diabetes prevention program coaches: a pilot study Internet. Research Square.

[58] Jung ME, Bourne JE, Beauchamp MR, Robinson E, Little JP (2015). High-intensity interval training as an efficacious alternative to moderate-intensity continuous training for adults with prediabetes. J Diabetes Res.

[59] Locke SR, Bourne JE, Beauchamp MR, Little JP, Barry J, Singer J, Jung ME (2018). High-intensity interval or continuous moderate exercise: a 24-week pilot trial. Med Sci Sports Exercise.

[60] Jung ME, Locke SR, Bourne JE, Beauchamp MR, Lee T, Singer J, MacPherson M, Barry J, Jones C, Little JP (2020). Cardiorespiratory fitness and accelerometer-determined physical activity following one year of free-living high-intensity interval training and moderate-intensity continuous training: a randomized trial. Int J Behav Nutr Phys Act.

[61] Curran GM, Bauer M, Mittman B, Pyne JM, Stetler C (2012). Effectiveness-implementation hybrid designs: combining elements of clinical effectiveness and implementation research to enhance public health impact. Med Care.

[62] MacPherson MM, Dineen TE, Cranston KD, Jung ME (2020). Identifying behaviour change techniques and motivational interviewing techniques in small steps for big changes: a community-based program for adults at risk for type 2 diabetes. Can J Diabetes.

[63] Cranston KD, Grieve NJ, Dineen TE, Jung ME (2024). Designing and developing online training for diabetes prevention program coaches using an integrated knowledge translation approach: development and usability study. JMIR Form Res.

[64] Grieve NJ, Cranston KD, Jung ME (2024). Examining the effectiveness of an e-learning training course for coaches of a type 2 diabetes prevention program. J Technol Behav Sci.

[65] Hsieh HF, Shannon SE (2005). Three approaches to qualitative content analysis. Qual Health Res.

[66] Wiltsey Stirman S, Baumann AA, Miller CJ (2019). The FRAME: an expanded framework for reporting adaptations and modifications to evidence-based interventions. Implement Sci.

[67] Damschroder LJ, Reardon CM, Widerquist MAO, Lowery J (2022). The updated consolidated framework for implementation research based on user feedback. Implement Sci.

[68] Waltz TJ, Powell BJ, Fernández ME, Abadie B, Damschroder LJ (2019). Choosing implementation strategies to address contextual barriers: diversity in recommendations and future directions. Implement Sci.

[69] Miller CJ, Barnett ML, Baumann AA, Gutner CA, Wiltsey-Stirman S (2021). The FRAME-IS: a framework for documenting modifications to implementation strategies in healthcare. Implement Sci.

[70] Shea CM, Jacobs SR, Esserman DA, Bruce K, Weiner BJ (2014). Organizational readiness for implementing change: a psychometric assessment of a new measure. Implement Sci.

[71] McLean KA (2013). Healthcare provider acceptability of a behavioral intervention to promote adherence - University of Miami. University of Miami.

[72] Weiner BJ, Lewis CC, Stanick C, Powell BJ, Dorsey CN, Clary AS, Boynton MH, Halko H (2017). Psychometric assessment of three newly developed implementation outcome measures. Implement Sci.

[73] Sekhon M, Cartwright M, Francis JJ (2022). Development of a theory-informed questionnaire to assess the acceptability of healthcare interventions. BMC Health Serv Res.

[74] (2021). Net Promoter Score. Project Management Institute.

[75] Vossen J, Burduli E, Barbosa-Leiker C (2017). MICA Manual.

[76] Powell BJ, Waltz TJ, Chinman MJ, Damschroder LJ, Smith JL, Matthieu MM, Proctor EK, Kirchner JE (2015). A refined compilation of implementation strategies: results from the expert recommendations for implementing change (ERIC) project. Implement Sci.

[77] Braun V, Clarke V, Hayfield N, Terry G (2019). Handbook of research methods in health social sciences. Thematic Analysis.

[78] Tracy SJ (2010). Qualitative quality: eight “big-tent” criteria for excellent qualitative research. Qual Inq.

[79] Rikli RE, Jones CJ (1998). The reliability and validity of a 6-minute walk test as a measure of physical endurance in older adults. J Aging Phys Act.

[80] Comino EJ, Tran DT, Haas M, Flack J, Jalaludin B, Jorm L, Harris MF (2013). Validating self-report of diabetes use by participants in the 45 and up study: a record linkage study. BMC Health Serv Res.

[81] Margolis KL, Lihong Q, Brzyski R, Bonds DE, Howard BV, Kempainen S, Simin L, Robinson JG, Safford MM, Tinker LT, Phillips LS, Women Health Initiative Investigators (2008). Validity of diabetes self-reports in the Women's Health Initiative: comparison with medication inventories and fasting glucose measurements. Clin Trials.

[82] Herdman M, Gudex C, Lloyd A, Janssen M, Kind P, Parkin D, Bonsel G, Badia X (2011). Development and preliminary testing of the new five-level version of EQ-5D (EQ-5D-5L). Qual Life Res.

[83] Godin G, Shephard RJ (1985). Leisure-time exercise questionnaire. APA PsycTests.

[84] (2021). Physical activity vital sign. American College of Sports Medicine.

[85] Ross R, Chaput JP, Giangregorio LM, Janssen I, Saunders TJ, Kho ME, Poitras VJ, Tomasone JR, El-Kotob R, McLaughlin EC, Duggan M, Carrier J, Carson V, Chastin SF, Latimer-Cheung AE, Chulak-Bozzer T, Faulkner G, Flood SM, Gazendam MK, Healy GN, Katzmarzyk PT, Kennedy W, Lane KN, Lorbergs A, Maclaren K, Marr S, Powell KE, Rhodes RE, Ross-White A, Welsh F, Willumsen J, Tremblay MS (2020). Canadian 24-hour movement guidelines for adults aged 18-64 years and adults aged 65 years or older: an integration of physical activity, sedentary behaviour, and sleep. Appl Physiol Nutr Metab.

[86] Block G, Azar KMJ, Romanelli RJ, Block TJ, Palaniappan LP, Dolginsky M, Block CH (2016). Improving diet, activity and wellness in adults at risk of diabetes: randomized controlled trial. Nutr Diabetes.

[87] Raes F, Pommier E, Neff KD, Van Gucht D (2011). Construction and factorial validation of a short form of the Self-Compassion Scale. Clin Psychol Psychother.

[88] Maetzel A, Li LC, Pencharz J, Tomlinson G, Bombardier C (2004). The economic burden associated with osteoarthritis, rheumatoid arthritis, and hypertension: a comparative study. Ann Rheum Dis.

[89] Guyatt GH, Sullivan MJ, Thompson PJ, Fallen EL, Pugsley SO, Taylor DW, Berman LB (1985). The 6-minute walk: a new measure of exercise capacity in patients with chronic heart failure. Can Med Assoc J.

[90] Bansback N, Tsuchiya A, Brazier J, Anis A (2012). Canadian valuation of EQ-5D health states: preliminary value set and considerations for future valuation studies. PLoS One.

[91] Drummond M, Manca A, Sculpher M (2005). Increasing the generalizability of economic evaluations: Recommendations for the design, analysis, and reporting of studies. Int J Technol Assess Health Care.

[92] Jackson MC, Dai S, Skeete RA, Owens-Gary M, Cannon MJ, Smith BD, Jabrah R, Masalovich SE, Soler RE (2020). An examination of gender differences in the National Diabetes Prevention Program’s lifestyle change program. Diabetes Educ.

[93] Pinnock H, Barwick M, Carpenter CR, Eldridge S, Grandes G, Griffiths CJ, Rycroft-Malone J, Meissner P, Murray E, Patel A, Sheikh A, Taylor SJC (2017). Standards for Reporting Implementation Studies (StaRI) statement. BMJ.

[94] Ely EK, Gruss SM, Luman ET, Gregg EW, Ali MK, Nhim K, Rolka DB, Albright AL (2017). A national effort to prevent type 2 diabetes: participant-level evaluation of CDC's national diabetes prevention program. Diabetes Care.

[95] Valabhji J, Barron E, Bradley D, Bakhai C, Fagg J, O'Neill S, Young B, Wareham N, Khunti K, Jebb S, Smith J (2020). Early outcomes from the English National Health Service Diabetes Prevention Programme. Diabetes Care.

[96] Lavis JN, Robertson D, Woodside JM, McLeod CB, Abelson J, Knowledge Transfer Study Group (2003). How can research organizations more effectively transfer research knowledge to decision makers?. Milbank Q.

[97] Graham ID, Logan J, Harrison MB, Straus SE, Tetroe J, Caswell W, Robinson N (2006). Lost in knowledge translation: time for a map?. J Contin Educ Health Prof.

[98] Grimshaw JM, Eccles MP, Lavis JN, Hill SJ, Squires JE (2012). Knowledge translation of research findings. Implement Sci.

