# Supplementary material for: Scaling Up a Diabetes Prevention Program in Geographically and Ethnoculturally Diverse Urban Regions of Canada: Protocol for a Hybrid Type 2 Implementation-Effectiveness Study
Source: JMIR Res Protoc. 2026 Jan 19;15:e80276. doi: 10.2196/80276 (PMC12865348; doi:10.2196/80276)
Supplement: Multimedia Appendix 1 [file resprot_v15i1e80276_app1.pdf]

November/Novembre 09, 2022

DC0190GP

Canadian Institutes of Health Research / Instituts de recherche en santé du Canada

Notice of Decision / Avis de décision

Application Number/Numéro de la demande: 485651
Committee Code/Code du comité: HTG

Applicant/Candidat: Dr. Mary E Jung

Institution paid/ University of British Columbia

Title/Titre: Small Steps for Big Changes: Implementing an Evidence-Based Diabetes Prevention Program into Diverse Urban Communities

Primary Inst./ Inst. principal: Nutrition, Metabolism and Diabetes / Nutrition, métabolisme et diabète

Other Related Inst./ Autres inst. connexes:

Competition Outcome/Résultats du concours: Team Grant : Healthy Cities Implementation Science (HCIS) Team Grants / Subvention d'équipe : Subventions d'équipe en SMO – domaine des villes en santé
July/Juillet 6, 2022

Number in competition/Nbre de demandes dans le concours: 15

Number approved/Nbre de demandes approuvées: 6

Decision on your application/ Décision sur votre demande: Approved / Approuvée   Other offer / Autre offre

Average annual amount/ Montant annuel moyen: $0

Term/Durée: **6 yrs/ans 0 months/mois**

Peer Review Committee Recommendation, for your information and use/
Recommandation du comité d'examen par les pairs, pour fins d'information et d'utilisation:

Committee/Comité: Team Grant : Healthy Cities Implementation Science (HCIS) Team Grants / Subvention d'équipe : Subventions d'équipe en SMO – domaine des villes en santé

Application rank within the competition/ 3

Percent Rank Within the Competition/ 20.00%

Rating/ 4.43

Recommended average annual amount/ Montant annuel moyen recommandé: $2,958,703

Additional Funding Opportunities/ Opportunités de financement add	Decision/ Décision	Total Funding Amount/Montant total du financement	Term/ Terme	Competition Code/Cote de conc	Application Number/ Numéro de la demande
Team Grant: HCIS - Type 2 diabetes prevention/Subv. d'équipe : SMOVS - Prévention du diabète de type 2	Approved/ Approuvée	$2,958,703	6 yrs/ans 0 mth/mois	202207HG4	489176

*** Applications receiving a score of less than 3.5 on any evaluation criteria will not be considered for Funding. / Les demandes qui ont reçu une note inférieure à 3.5 pour n'importe quel des critères d'évaluation ne sont pas admissibles.

This document is for information only. Official payment is stated on the CIHR Authorization for Funding.
Document à titre d'information seulement. Le paiement officiel est indiqué sur l'autorisation de financement des IRSC.

Canadian Institutes of Health Research
160 Elgin Street, 9th Floor
Address Locator 4809A
Ottawa, Ontario K1A 0W9

Instituts de recherche en santé du Canada
160, rue Elgin, 9e étage
Indice de l'adresse 4809A
Ottawa (Ontario) K1A 0W9

Institute of Aging
Institute of Cancer Research
Institute of Circulatory and Respiratory Health
Institute of Gender and Health
Institute of Genetics
Institute of Health Services and Policy Research
Institute of Human Development, Child and Youth Health
Institute of Indigenous Peoples' Health
Institute of Infection and Immunity
Institute of Musculoskeletal Health and Arthritis
Institute of Neurosciences, Mental Health and Addiction
Institute of Nutrition, Metabolism and Diabetes
Institute of Population and Public Health

Institut du vieillissement
Institut du cancer
Institut de la santé circulatoire et respiratoire
Institut de la santé des femmes et des hommes
Institut de génétique
Institut des services et des politiques de la santé
Institut du développement et de la santé des enfants et des adolescents
Institut de la santé des Autochtones
Institut des maladies infectieuses et immunitaires
Institut de l'appareil locomoteur et de l'arthrite
Institut des neurosciences, de la santé mentale et des toxicomanies
Institut de la nutrition, du métabolisme et du diabète
Institut de la santé publique et des populations

November 9, 2022

Dr. Mary Jung
University of British Columbia Okanagan
School of Health and Exercise Sciences
Health Sciences Centre room 119
3333 University Way
Kelowna, British Columbia
V1V 1V7
Canada

Dear Dr. Jung,

On behalf of the Canadian Institutes of Health Research (CIHR), and we are pleased to inform you that your recent application submitted to the Team Grant : Healthy Cities Implementation Science (HCIS) Team Grants - Full Application competition, has been approved for funding by CIHR. Your application for supplemental funds from the National Health and Medical Research Council (NHMRC) has also been approved for funding. Your results from the Public Health Agency of Canada (PHAC) component of your application will be sent in a separate Notice of Decision directly from PHAC at a later date.

Documentation pertaining to the review of your application can be found through ResearchNet. Please note that your Authorization for Funding will follow.

As CIHR does not notify co-applicants of the decision, we ask that you inform those individuals involved, along with their research institutions (if different from your own), of the outcome of this application.

Should you require additional information, please contact the CIHR Contact Centre at support@cihr-irsc.gc.ca. Please do not contact the officers or members of the peer review committee. Should you have questions pertaining to the outcome of the PHAC component of your application, please contact hccf-fscc@phac-aspc.gc.ca.

Congratulations on your success in this competition.

Sincerely,

C. Leneis

Chaidwick Leneis

Canada

CIHR IRSC
Discoveries for life / Découvertes pour la vie

Manager, Program Design and Delivery
Research Programs Portfolio

Canadian Institutes of Health Research
160 Elgin Street, 9th Floor
Address Locator 4809A
Ottawa, Ontario K1A 0W9

Instituts de recherche en santé du Canada
160, rue Elgin, 9e étage
Indice de l'adresse 4809A
Ottawa (Ontario) K1A 0W9

Institute of Aging
Institute of Cancer Research
Institute of Circulatory and Respiratory Health
Institute of Gender and Health
Institute of Genetics
Institute of Health Services and Policy Research
Institute of Human Development and Child and Youth Health
Institute of Indigenous Peoples' Health
Institute of Infection and Immunity
Institute of Musculoskeletal Health and Arthritis
Institute of Neurosciences, Mental Health and Addiction
Institute of Nutrition, Metabolism and Diabetes
Institute of Population and Public Health

Institut du vieillissement
Institut du cancer
Institut de la santé circulatoire et respiratoire
Institut de la santé des femmes et des hommes
Institut de génétique
Institut des services et des politiques de la santé
Institut du développement et de la santé des enfants et des adolescents
Institut de la santé des Autochtones
Institut des maladies infectieuses et immunitaires
Institut de l'appareil locomoteur et de l'arthrite
Institut des neurosciences, de la santé mentale et des toxicomanies
Institut de la nutrition, du métabolisme et du diabète
Institut de la santé publique et des populations

November 9, 2022

Dr. Mary Elizabeth Jung
University of British Columbia Okanagan
School of Health and Exercise Sciences
1147 Research Road, ART360-FHSD
Reichwald Health Sciences Centre room 119
Kelowna, British Columbia V1V 1V7

Dear Dr. Jung:

On behalf of the Canadian Institutes of Health Research (CIHR), I wish to congratulate you on your success in the recent CIHR funding competition!

As health researchers, we are united by a common goal: to improve the health and well-being of Canadians and people throughout the world. Through CIHR, the Government of Canada provides vital support to Canadian researchers, spanning the tightly linked pillars of health research, with the ultimate goal of improving health for all Canadians. As a recipient of this funding, you are now part of this endeavour.

As you are aware, the evaluation of your grant application was made possible thanks to peer reviewers who generously volunteer their time to support the Canadian health research enterprise. As a CIHR-funded researcher, your knowledge and expertise are invaluable to this process, and I encourage you to participate in peer review activities when invited to do so. Additionally, if you have not already joined the College of Reviewers, please consider becoming a member. To learn more about selection criteria and how to apply for membership in the College of Reviewers, please visit www.cihr-irsc.gc.ca/e/49923.html.

As health researchers, we are living at a time when science is under careful scrutiny, and it has become more important than ever to ensure that our work is clearly understood. As such, I would ask that you recognize your CIHR funding when delivering presentations and communicating about your research, and that you continue to advocate for the critical importance of research in advancing the health of Canadians. Please visit https://cihr-irsc.gc.ca/e/30789.html to learn about ways to acknowledge funding and how the CIHR Communications team can help promote your research.

Once again, please accept my congratulations on this impressive achievement and my best wishes for success in all your endeavours. I look forward to following the progress of your research.

Sincerely,

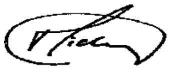

Michael J. Strong, MD, FRCPC, FAAN, FCAHS
President

507891-202207HTG-HTG-485651-I013-138478-CONGR

CIHR IRSC
Discoveries for life / Découvertes pour la vie

Canada

Review Type/Type d'évaluation: Committee Member 1/Membre de comité 1
Name of Applicant/Nom du chercheur: Jung, Mary Elizabeth
Application No./Numéro de demande: 485651
Agency/Agence: CIHR/IRSC
Competition/Concours: 2022-07-06 Team Grant: Healthy Cities Implementation Science (HCIS) Team Grants/Subventions d'équipe : Subventions d'équipe en science de la mise en œuvre dans le domaine des villes en santé (SMOVS)
Committee/Comité: Team Grant: Healthy Cities Implementation Science (HCIS) Team Grants/Subv. d'équipe: Subventions d'équipe en SMO - domaine des villes en santé
Title/Titre: Small Steps for Big Changes: Implementing an Evidence-Based Diabetes Prevention Program into Diverse Urban Communities

---

Assessment/Évaluation:

Summary:
The Small Steps for Big Changes (SSBC) is a 3-week evidence-based diabetes prevention program that includes diet and exercise counselling and can be delivered in the community. It has been previously shown to be highly effective in improving HBA1c and preventing Type II Diabetes among people with pre-diabetes. The team proposes to work with 11 Canadian and 2 Australian partners to implement SSBC in 16 urban communities (11 in Canada and 5 in Australia). The municipalities chosen were selected to ensure ethnocultural and socioeconomic diversity. The Interactive Systems Framework for Dissemination and Implementation and the Integrated Sustainability Framework will be used to guide the project. The team proposes a hybrid Type II implementation-effectiveness study design that will occur in 3 phases: pre-implementation phase (adaptation of the SSBC and developing recruitment plans), implementation phase (SSBC will be implemented in 11 YMCAs in Canada and 5 municipalities in Australia), and the sustainability phase (co-creation of a sustainability model). Both virtual and in-person modes of delivery will be offered, and participants will have access to the SSBC app. Partners are providing coach time as in-kind support.
Implementation outcomes include adoption, reach, dose, fidelity and sustainability, and these will be obtained from session audit data and checklists, focus groups, interviews, and questionnaires. There is a detailed plan for evaluating effectiveness using self-report tools via the SSBC app. A sub-sample of individuals will also have measurements of HBA1c, cardiorespiratory fitness and cost-effectiveness at baseline and then 1 and 2 years following the program.
Key research questions identified in the proposal include and examination of what program and implementation strategy adaptations are needed for SSBC to meet the needs of diverse individuals, and identifying the organizational, programmatic, and contextual factors that will optimize SSBC implementation and sustainability.

Strengths:
SSBC is an evidence-based program that has been shown to be remarkably effective in Phase I, II, and III trials already completed.
The SSBC modules include training in cultural sensitivity and inclusivity.
The research team is exceptionally strong with track records of success and the team includes committed community partners. Research environment will provide excellent training opportunities.
Proposal was co-developed by the research team, patient partners, and the PKUs. Research questions were iteratively refined and are of specific interest to the PKUs and community partners.
Analysis plan for the effectiveness outcomes is clear and detailed. Including cost-effectiveness is a strength of the proposal.
Plans for knowledge sharing are excellent; the Coach Community of Practice will help to build capacity among community partners.

**Review Type/Type d'évaluation:**	Committee Member 1/Membre de comité 1
**Name of Applicant/Nom du chercheur:**	Jung, Mary Elizabeth
**Application No./Numéro de demande:**	485651
**Agency/Agence:**	CIHR/IRSC
**Competition/Concours:**	2022-07-06 Team Grant: Healthy Cities Implementation Science (HCIS) Team Grants/Subventions d'équipe : Subventions d'équipe en science de la mise en œuvre dans le domaine des villes en santé (SMOVS)
**Committee/Comité:**	Team Grant: Healthy Cities Implementation Science (HCIS) Team Grants/Subv. d'équipe: Subventions d'équipe en SMO - domaine des villes en santé
**Title/Titre:**	Small Steps for Big Changes: Implementing an Evidence-Based Diabetes Prevention Program into Diverse Urban Communities

Assessment/Évaluation:

Weaknesses:
Not entirely clear on the rationale for a Type 2 Hybrid design. The rationale for the project is very convincing that this program is effective and that implementation and scale-up is where the knowledge gap lies. If so, then why not focus more explicitly on implementation outcomes as opposed to having co-primary aims? A greater focus on implementation would address some of the concerns outlined below.
Although the research objectives related to implementation are excellent, the analysis plan for the implementation outcomes is very vague, especially in comparison to the analysis plan for the effectiveness outcomes. For example, one key goal is to examine program and implementation strategy adaptations and how they meet the needs of diverse individuals, but it is not clear how this will be achieved. If each of the 16 sites can adapt the program as they see fit and there are different contextual factors in each site, what is the plan to identify which factors and adaptations affect implementation? There is some vague language about data being "analyzed descriptively" and thematic analyses, but overall, it is not clear how the list of "best practices and resources" will be determined.
Specific roles of the research team members are not clearly defined.
The focus on equity is important, but the plans to "meet the needs of diverse sex, gender, municipal, and ethnocultural contexts" are not well defined. Is there any information on ethnocultural identity and success of the program that can be drawn from previous trials of the SSBC? Although the implementation sites have ostensibly been chosen specifically because of "access to diverse and underserved populations, the threshold for "diversity" and definition of "access" is not clear. For the effectiveness evaluation there is a plan to oversample participants who are in underrepresented groups, which is very broadly defined as not identifying as man or White. But what are the plans to ensure the total participants include underrepresented groups? There is mention of developing a recruitment plan but the target audience isn't clear. Are there any plans to partner with community organizations that serve specific underserved groups? This component of the proposal feels too broad and too vague to be convincing.

Budget
Overall the budget seems reasonable and aligned with the proposal. Some budget items could be considered part of the intervention- e.g. time for coach training for program delivery, train-the-trainer platform, SSBC Platform.
Sex and gender considerations
Sex and gender will be assessed using a survey that has been pilot tested by the research team. All data will be disaggregated by sex and gender for both implementation and effectiveness outcomes. Women are more likely to participate in prevention and health promotion programs, but participation of different sexes and genders in past trials of SSBC is not identified. The proposal specifically suggests that to focus on

**Review Type/Type d'évaluation:**	Committee Member 1/Membre de comité 1
**Name of Applicant/Nom du chercheur:**	Jung, Mary Elizabeth
**Application No./Numéro de demande:**	485651
**Agency/Agence:**	CIHR/IRSC
**Competition/Concours:**	2022-07-06 Team Grant: Healthy Cities Implementation Science (HCIS) Team Grants/Subventions d'équipe : Subventions d'équipe en science de la mise en œuvre dans le domaine des villes en santé (SMOVS)
**Committee/Comité:**	Team Grant: Healthy Cities Implementation Science (HCIS) Team Grants/Subv. d'équipe: Subventions d'équipe en SMO - domaine des villes en santé
**Title/Titre:**	Small Steps for Big Changes: Implementing an Evidence-Based Diabetes Prevention Program into Diverse Urban Communities

Assessment/Évaluation:

underrepresented groups they will oversample participants who are not male. This seems incongruent with the fact that males are more likely have diabetes and women are more likely to participate in a DPP.

PHAC

PHAC funding will be used to scale up over the last 5 years of the CIHR project – beyond the 11 YMCA sites in the CIHR proposal, they will scale up to offer SSBC in all of the remaining 33 YMCAs in urban sites. This would increase the potential impact of the project on health and implementation science. No new questions, just larger expansion with same design. I could not find a budget for the PHAC proposal?

**Review Type/Type d'évaluation:**	Committee Member 2/Membre de comité 2
**Name of Applicant/Nom du chercheur:**	Jung, Mary Elizabeth
**Application No./Numéro de demande:**	485651
**Agency/Agence:**	CIHR/IRSC
**Competition/Concours:**	2022-07-06 Team Grant: Healthy Cities Implementation Science (HCIS) Team Grants/Subventions d'équipe : Subventions d'équipe en science de la mise en œuvre dans le domaine des villes en santé (SMOVS)
**Committee/Comité:**	Team Grant: Healthy Cities Implementation Science (HCIS) Team Grants/Subv. d'équipe: Subventions d'équipe en SMO - domaine des villes en santé
**Title/Titre:**	Small Steps for Big Changes: Implementing an Evidence-Based Diabetes Prevention Program into Diverse Urban Communities

Assessment/Évaluation:

1. Research approach – Small steps for big changes is a diet and exercise counselling intervention that decreases risk for Type 2 Diabetes. Program allows for peer delivered approach to enhance ethnocultural sensitivity. This evidence based intervention has been tested, is now available virtually using a state of the art online platform and ready to be deployed nationally to culturally diverse groups using peer coaches. Plan to test the implementation of the program in areas where there is high T2D and low accessibility. Partnership with YMCA in Canada and Australia across 8 provinces (16 urban Municipalities) & Queensland. Nine research questions related to implementation approaches, contexts, impacts and effectiveness.
2. Applicants – very strong team of 38 researchers - Principle applicant is a recognized expert in sex and gender and the plan for Sex and Gender considerations is well described. Excellent collaborations with YMCA across Canada and Internationally. Committed to providing in kind staff to support program delivery and recruitment of community members / participants. Good blend of early, mid and senior researchers.
3. Environment for the Research – the vision and approach is to reduce the incidence of T2Dacross Canada and around the globe. The integrated sustainability Framework and Interactive System Framework for dissemination and implementation used to understand and describe the dynamic nature of implementing a DPP into real world contexts. A hybrid Type 2 implementation effectiveness study design used to evaluate co-aims of implementation and effectiveness of SSBC. Three phase plan for implementation
4. Impact of the Research – Goal is to significantly reduce T2D via introduction of SSBC. Given the rise in T2D in the population and that is preventable, this research has the potential to have a significant impact on the health of the populations and reduction in health service utilization. To achieve this objective, the implementation plan aims to scale across the country to have a population impact, while contributing to the IS in multiple heterogenous communities. The partnerships with YMCAs achieves this and project meets the objectives of the T2D theme.

Strengths;
- Evidence Based intervention with an opportunity to customize to vulnerable community populations using Peer Coaches for ethnocultural customization
- Implementation plan is well articulated
- Impact across 8 provinces and Australia – with 16 sites in total
- Excellent collaboration with partner organizations – YMCA – strong in kind support
- YMCA has excellent connection to the communities they serve so customizing the program to constituents of the community

**Review Type/Type d'évaluation:**	Committee Member 2/Membre de comité 2
**Name of Applicant/Nom du chercheur:**	Jung, Mary Elizabeth
**Application No./Numéro de demande:**	485651
**Agency/Agence:**	CIHR/IRSC
**Competition/Concours:**	2022-07-06 Team Grant: Healthy Cities Implementation Science (HCIS) Team Grants/Subventions d'équipe : Subventions d'équipe en science de la mise en œuvre dans le domaine des villes en santé (SMOVS)
**Committee/Comité:**	Team Grant: Healthy Cities Implementation Science (HCIS) Team Grants/Subv. d'équipe: Subventions d'équipe en SMO - domaine des villes en santé
**Title/Titre:**	Small Steps for Big Changes: Implementing an Evidence-Based Diabetes Prevention Program into Diverse Urban Communities

Assessment/Évaluation:

- Selection of sites where there is high T2D prevention capability and with a focus on vulnerable groups and low access
- Significant contributions to Implementation Science in communities
- Strong team, Sex and Gender expertise as it relates to iKT
- Patient Partners part of the design, research questions, analytic approaches and dissemination strategies

Weaknesses:

- Potential difficulty with recruitment
- Could strengthen collaboration with primary care and their role in follow up

Review Type/Type d'évaluation: Committee Member 3/Membre de comité 3
Name of Applicant/Nom du chercheur: Jung, Mary Elizabeth
Application No./Numéro de demande: 485651
Agency/Agence: CIHR/IRSC
Competition/Concours: 2022-07-06 Team Grant: Healthy Cities Implementation Science (HCIS) Team Grants/Subventions d'équipe : Subventions d'équipe en science de la mise en œuvre dans le domaine des villes en santé (SMOVS)
Committee/Comité: Team Grant: Healthy Cities Implementation Science (HCIS) Team Grants/Subv. d'équipe: Subventions d'équipe en SMO - domaine des villes en santé
Title/Titre: Small Steps for Big Changes: Implementing an Evidence-Based Diabetes Prevention Program into Diverse Urban Communities

---

Assessment/Évaluation:

Small Steps for Big Changes: Implementing an Evidence-Based Diabetes Prevention Program into Diverse Urban Communities

Summary
This project proposes to scale and expand a brief, online diabetes prevention program (Small Steps for Big Changes, SSBC) delivered by lay people in the community to 9 regional YMCAs across Canada. The program is the culmination of eight years of work by the primary applicant to establish and demonstrate the efficacy and effectiveness of the program. The proposal includes both implementation and effectiveness questions which had been co-developed with partners. Implementation questions are as follows: (1) What strategies are used to reach targeted audiences and what municipal factors influence these? (2) What program and implementation strategy adaptations are needed for SSBC to meet the needs of diverse sex, gender, municipal, and ethnocultural contexts? (3) What implementation determinants influence implementation outcomes, in what ways, and under what circumstances? (4) How does ethnocultural identity, sex, and gender and (mis)matches of these between patients and coaches intersect with program delivery, satisfaction, engagement, and effectiveness outcomes? (5) What organizational, programmatic, and contextual factors optimize SSBC implementation and sustainability? (6) What program components are sustained or adapted over time, and what influence do adaptations have on sustainability? And the effectiveness questions built into the study include: (7) What is the long-term impact of SSBC on diabetes status, physical activity, diet, weight, and cardiorespiratory fitness in individuals with prediabetes? (8) What is the cost-effectiveness of SSBC? (9) How does implementation impact clinical- and cost-effectiveness of SSBC across distinct settings?

Strengths of this application are as follows:

Research approach
There is a strong rationale for the need to address the ever-growing population of Canadians with diabetes (or pre-diabetes). The proposed intervention is evidence-based and acceptable to partners given the ease in which it can be implemented in real-world settings and the (relatively) low cost. The proposed blended methodology is efficient and will contribute to both implementation science and health outcomes research. This is one of the few proposals I reviewed with implementation science questions first and foremost plus very detailed measures, so it aligns with the goals of the funding call to advance the field and build capacity.
Applicants
This is an excellent research team with content expertise (diabetes researchers and those with lived experience, gender champions, implementation science experts etc.). There are also early, mid and senior career level applicants. The applicants have an extensive record of successful grants and scholarly achievement.

**Review Type/Type d'évaluation:**	Committee Member 3/Membre de comité 3
**Name of Applicant/Nom du chercheur:**	Jung, Mary Elizabeth
**Application No./Numéro de demande:**	485651
**Agency/Agence:**	CIHR/IRSC
**Competition/Concours:**	2022-07-06 Team Grant: Healthy Cities Implementation Science (HCIS) Team Grants/Subventions d'équipe : Subventions d'équipe en science de la mise en œuvre dans le domaine des villes en santé (SMOVS)
**Committee/Comité:**	Team Grant: Healthy Cities Implementation Science (HCIS) Team Grants/Subv. d'équipe: Subventions d'équipe en SMO - domaine des villes en santé
**Title/Titre:**	Small Steps for Big Changes: Implementing an Evidence-Based Diabetes Prevention Program into Diverse Urban Communities

Assessment/Évaluation:

Environment for the Research
This is a well established PI and research team with the necessary infrastructure to deliver on the project and a strong track record of mentoring students across disciplines and academic levels.
Impact of the Research
This was one of the few proposals that took an equity lens and also is studying implementation in many settings (11 Canadian and 5 Australian urban municipalities) which increases the potential for impact. The potential reach of those most impacted by diabetes is promising.

Weaknesses noted in the application:
Light on details around the implementation analyses and tracking all the potential modifications to the core program, perhaps worth considering core elements.
Budget
All seems appropriate and detailed justification is provided. Significant investment in trainees over the 6 year grant.

Review Type/Type d'évaluation: Committee Member 4/Membre de comité 4
Name of Applicant/Nom du chercheur: Jung, Mary Elizabeth
Application No./Numéro de demande: 485651
Agency/Agence: CIHR/IRSC
Competition/Concours: 2022-07-06 Team Grant: Healthy Cities Implementation Science (HCIS) Team Grants/Subventions d’équipe : Subventions d’équipe en science de la mise en œuvre dans le domaine des villes en santé (SMOVS)
Committee/Comité: Team Grant: Healthy Cities Implementation Science (HCIS) Team Grants/Subv. d’équipe: Subventions d’équipe en SMO - domaine des villes en santé
Title/Titre: Small Steps for Big Changes: Implementing an Evidence-Based Diabetes Prevention Program into Diverse Urban Communities

---

Assessment/Évaluation:

1. Research approach

Strengths: Strong implementation science approach; Evidence-based diabetes prevention program with high potential for scalability; Proposed work builds upon program of research developed and expanded methodically over past decade; Very strong partnership with YMCA (which provides excellent potential for successful implementation and future scalability) and municipalities; Planned implementation across 16 urban municipalities selected to maximize ethnocultural, population, and socioeconomic diversity; Concrete plan for pre-implementation/implementation/sustainability phases;

Weaknesses: All efficacy data will be based on program participants (no comparison group)

2. Applicants

Strengths: NPA has strong track record as PI on grants related to the present proposal; NPA has strong publication track record

Weaknesses: none

3. Environment for the Research

Strengths: Excellent research environment for NPA

Weaknesses: none

4. Impact of the Research

Strengths: High potential for impact given proven intervention, strong implementation science approach and potential for scale up through partnership with YMCA sites across the country

Weaknesses: none

Review Type/Type d'évaluation: SO Notes /Notes de l'agent scientifique
Name of Applicant/Nom du chercheur: Jung, Mary Elizabeth
Application No./Numéro de demande: 485651
Agency/Agence: CIHR/IRSC
Competition/Concours: 2022-07-06 Team Grant: Healthy Cities Implementation Science (HCIS) Team Grants/Subventions d’équipe : Subventions d’équipe en science de la mise en œuvre dans le domaine des villes en santé (SMOVS)
Committee/Comité: Team Grant: Healthy Cities Implementation Science (HCIS) Team Grants/Subv. d’équipe: Subventions d’équipe en SMO - domaine des villes en santé
Title/Titre: Small Steps for Big Changes: Implementing an Evidence-Based Diabetes Prevention Program into Diverse Urban Communities

---

Assessment/Évaluation:

Strengths:

This is a very well written proposal. The project is based on an evidence-based intervention that has shown to be effective and has benefited from past trials and scale up. This is a strong team with a highly experienced NPA. There are good guidelines to implement the project. Site selection and YWCA partnerships will increase diversity of the participants and will be an excellent pathway to broader implementation. YMCA support in-kind is significant and could increase the use of peer counsellors. The co-creation of sustainability plans with partners and patient partnerships that will support project codesign are both strengths. There is an excellent training environment that includes training in cultural sensitivity and inclusion. There is a good knowledge sharing plan. The dispersion of study locations ion 8 provinces provides opportunities to examine local challenges in implementation. The project committee attending to diversity and inclusion will be helpful.

The project addresses PHAC target areas. PHAC investment will enable a wider impact over a broader area. The use of a proven method to prevent a rapidly accelerating problem is a strength.

Weaknesses:

Potential recruitment challenges were noted. If this proposal aims to reach underserviced people yet inclusion requires clinical testing, how will people underserved by clinical care know they are eligible. A stronger connection to primary care would benefit the project and the participants. If males are more likely to be diabetic and less likely to participant in health promotion or prevention programs, what is the rationale to emphasize non-male participants.
The analytical plan for the implementation science research was sparse, making it hard to see how best practices will be revealed. Definitions of what is meant by diversity and access will help better define the target groups. Partnerships with community groups that support underserved groups could improve inclusion of the desired targets groups. A description of how participants will be connected to primary care for recruitment and patient support was lacking in the proposal but suggested in letters of support.

Budget:

**Review Type/Type d'évaluation:**	SO Notes /Notes de l'agent scientifique
**Name of Applicant/Nom du chercheur:**	Jung, Mary Elizabeth
**Application No./Numéro de demande:**	485651
**Agency/Agence:**	CIHR/IRSC
**Competition/Concours:**	2022-07-06 Team Grant: Healthy Cities Implementation Science (HCIS) Team Grants/Subventions d’équipe : Subventions d’équipe en science de la mise en œuvre dans le domaine des villes en santé (SMOVS)
**Committee/Comité:**	Team Grant: Healthy Cities Implementation Science (HCIS) Team Grants/Subv. d’équipe: Subventions d’équipe en SMO - domaine des villes en santé
**Title/Titre:**	Small Steps for Big Changes: Implementing an Evidence-Based Diabetes Prevention Program into Diverse Urban Communities

Assessment/Évaluation:

CIHR to look at eligibility of proposed budget items
